# PLA, PBS, and PBAT Biocomposites—Part A: Matrix–Filler Interactions with Agro-Industrial Waste Fillers (Brewer’s Spent Grain, Orange Peel) and Their Influence on Thermal, Mechanical, and Water Sorption Properties

**DOI:** 10.3390/ma18163867

**Published:** 2025-08-18

**Authors:** Jules Bellon, Feriel Bacoup, Stéphane Marais, Richard Gattin

**Affiliations:** 1 UniLaSalle, Université Artois, ULR7519—Transformations & Agro-Ressources, Normandie Université, 3 Rue du Tronquet, 76130 Mont-Saint-Aignan, France; feriel.bacoup@unilasalle.fr (F.B.);; 2 Université Rouen Normandie, INSA Rouen Normandie, CNRS, Normandie Université, PBS UMR 6270, 76000 Rouen, France; stephane.marais@univ-rouen.fr

**Keywords:** biocomposites, biodegradable thermoplastics, agro-industrial wastes, circular economy, mechanical properties, thermal properties, water behavior, PLA, PBS, PBAT

## Abstract

Plastic pollution, largely driven by packaging waste, calls for sustainable alternatives. This study investigates biodegradable thermoplastic biocomposites based on PLA, PBS, and PBAT, incorporating 10 wt.% of agro-industrial filler-brewers’ spent grain (BSG) and orange peel (OP) without compatibilization. The biocomposites were produced by melt extrusion followed by thermo-compression. A full factorial design was implemented to assess matrix–filler interactions and compare biocomposites to pure polymer fragments. OP particles, smaller and rougher than BSG, exhibited a higher specific surface area, influencing composite morphology and behavior. The OP slightly plasticized PLA, possibly due to volatile release during processing, whereas BSG increased stiffness in PBS and PBAT. Both fillers reduced mechanical strength, especially in PLA, due to limited interfacial adhesion, and significantly decreased PLA’s thermal stability. The addition of fillers also increased water sorption and modified the sorption kinetics of the three main modes (Langmuir-type, Henry’s law sorption, and water molecule clustering), as well as the values of the half-sorption diffusion coefficients (D_1_ and D_2_), with notable differences between the OP and BSG linked to their structure and composition. These findings provide a better understanding of structure–property relationships in biodegradable composites and highlight their potential for sustainable packaging and other industrial applications.

## 1. Introduction

In recent years, growing environmental concerns have underscored the importance of biodegradable and/or bio-based polymers in the manufacturing of plastic products [1]. Conventional non-biodegradable petroleum-based polymers (e.g., PE, PET, PP) contribute significantly to pollution in all biospheric compartments, occurring at two critical stages of their life cycle: (i) the consumption of fossil resources during production and transportation, and (ii) the accumulation and persistence of post-consumer waste within ecosystems [2].

The packaging industry, which accounts for the largest share of global plastic consumption, is particularly targeted for sustainable alternatives. In this context, the circular economy approach offers a conceptual framework that promotes innovation by integrating environmental and societal performance with economic efficiency [3]. Achieving an optimal balance between these three aspects is essential to ensure sustainability at multiple scales.

Despite a notable increase in production in the past decade, the use of biobased and/or biodegradable polymers remains limited, representing only 0.5% of global plastic production in 2019 [4]. Projections suggest that their production will double by 2028 [5], yet their adoption is constrained by their higher cost compared to conventional petroleum-based plastics [6]. This price gap is mainly attributed to the low market price of petroleum, the complexity of processing natural resources for biopolymer synthesis, and the relatively small scale of current biopolymer production facilities, which have not yet achieved the economies of scale required for cost competitiveness [7]. Among the biodegradable polymers available on the market, polylactic acid (PLA) represents the largest share (40.1%), followed by starch-based blends (34.7%), polybutylene adipate-co-terephthalate (PBAT) (8.7%), polyhydroxyalkanoates (PHAs) (7.5%), cellulose derivatives (6.9%), and polybutylene succinate (PBS) (1.7%) [8].

Composite plastic materials offer significant advantages over pure polymer resins, particularly for packaging applications, where mechanical strength, thermal stability, gas barrier properties, and biodegradability must be optimized [9]. Their performance can be fine-tuned by selecting appropriate matrix-filler combinations, adjusting component ratios, and optimizing processing conditions. In addition, composites are often more cost-effective, as fillers are generally less expensive than polymer resins [10]. The production of composites using melt-processing techniques, such as thermo-extrusion and injection molding, is widely applied to thermoplastics and is considered economically viable and environmentally preferable to solution-based approaches [11].

In this context, the nature and origin of the filler play a crucial role in determining composite properties. While synthetic fillers have long dominated the field, growing attention is now being directed toward renewable biobased alternatives.

During the past decade, natural fibers (e.g., kenaf, jute, hemp, lyocell) have increasingly replaced synthetic fibers (e.g., glass fibers) to create lightweight, low-cost, and environmentally friendly biocomposites with a reduced environmental footprint [12]. Beyond natural fibers, organic fillers derived from agro-industrial waste and by-products are gaining attention due to their abundant availability and low economic value [13,14]. Biochemically, these fillers primarily consist of lignin, cellulose, hemicellulose, mono- and disaccharides, proteins, and phenolic compounds [15], imparting strong hydrophilic and polar characteristics. These agro-industrial wastes come from a wide variety of sources, including grass straws [16], bagasse from crops such as sugarcane and malt [17,18], fruit and vegetable peels [19], by-products of the dairy industry [20], crustacean shells [21], and grain or oilseed husks [22]. Due to their high moisture content, these materials often require drying and grinding before being incorporated into biocomposites [23]. Additional pre-treatments, such as acid or alkaline modifications, can be applied to enhance their compatibility with polymer matrices; however, these extra steps increase both the economic and environmental impact of biocomposite production, potentially diminishing the benefits of using low-value organic fillers [24].

Beer production, a major agro-industrial sector, generates an annual output ranging from 1.91 to 1.97 billion hectoliters [25]. A key by-product of brewing is brewers’ spent grain (BSG), which accounts for approximately 85% of solid residues generated [26]. BSG primarily consists of the insoluble grain fraction, including husks, pericarps, and hulls, mainly from barley but sometimes also from wheat, maize, or rice [27]. The brewing process generates approximately 20 kg of wet BSG per 100 L of beer produced, leading to an estimated annual global production of 38.6 million tons [28]. Chemically, BSG is composed of fibers (50% dry weight, including cellulose and hemicellulose), proteins (up to 30%), and lignin (10–30%) [29]. BSG is a promising functional filler for biodegradable packaging biocomposites, due to its low economic valorization, favorable chemical composition, and capacity to enhance stiffness and thermal stability through thermally induced transformations.

In this regard, Hejna et al. [30] reported a significant increase in elastic moduli—up to +174% in flexural and +139% in tensile strength (PN-EN ISO 527) [31]—in Mater-Bi^®^-based biocomposites (composed of PBAT, PHBV, and TPS) incorporating 30 wt.% of chemically functionalized brewer’s spent grain (BSG) using isophorone diisocyanate (IPDI). This functionalization aimed to reduce the polarity mismatch with the hydrophobic polyester components. The authors also demonstrated an increase in the onset temperature of thermal degradation (T_onset_) under oxidative conditions, attributed to the stabilizing effect of Maillard reaction products (i.e., melanoidins) generated during the twin-screw co-rotating extrusion process.

In a separate study, the same research group observed a more pronounced increase in surface roughness (248–448%) in biocomposites incorporating 30 wt.% BSG after soil burial, compared to the neat Mater-Bi^®^ matrix. This was assessed using atomic force microscopy (AFM) [32]. The increase in surface roughness was positively correlated with weight loss, indicating disintegration associated with material swelling and the formation of visible surface deformations.

Citrus fruits are the most consumed fruit group worldwide, with an annual production of 158 million tons [33], about half of which are oranges (Citrus sinensis). Juice processing generates large amounts of unused peels—around 20% of the fruit’s weight [34]—whose landfill disposal poses environmental challenges due to their low pH and bioactive compound content [35]. Biochemically, orange peels (OP) are rich in cellulose, hemicellulose, pectin, lignin, simple sugars, proteins, and flavonoids [19].

Several studies have investigated the incorporation of orange peel (OP) as a bio-based filler in biodegradable polyester matrices such as PLA, PBS, or PBSA. This strategy aims to enhance the biodegradability of the polymer matrix under mesophilic conditions (20–45 °C), impart bioactive properties to the resulting materials (e.g., bacteriostatic effects in low microbial density environments), and reduce production costs. For instance, Sambudi et al. [19] demonstrated that a high OP content (up to 60 wt.%) significantly increased the weight loss of PLA-based biocomposites in soil burial tests, although this improvement came at the expense of mechanical performance.

Koutoulis et al. [36] reported that the addition of citrus peel powders to PLA enhanced the antioxidant activity of the resulting films, with effects varying according to the botanical species of the citrus peels used. Pagliarini et al. [37] further confirmed that OP imparts antibacterial properties to PBSA due to its high content of phenolic compounds and may even improve the tensile strength of the biocomposite when incorporated at 15 wt.%. These findings highlight the potential of orange peel as a functional filler, although its effectiveness strongly depends on the interaction with the polymer matrix and the processing conditions used for biocomposite fabrication.

Given the importance of packaging as the primary sector driving plastic consumption, the incorporation of BSG and OP as fillers in biodegradable biocomposites represents a promising strategy to improve the sustainability of packaging materials. However, incorporating high amounts of these fillers may compromise thermomechanical performance due to poor interfacial adhesion between hydrophilic fillers and hydrophobic polymer matrices [38,39].

Despite these limitations, it has been shown that the biodegradability of biocomposites improves as the proportion of organic fillers increases, since polysaccharides and proteins are more susceptible to microbial degradation under mesophilic conditions. This is mainly explained by the fact that the preferential biodegradation of the filler induces the early disintegration of the biocomposite (i.e., formation of smaller particles), thereby increasing the polymer matrix surface area exposed to microbial hydrolytic enzymes and, consequently, accelerating the degradation rate [40,41].

This study presents a comparative analysis of the production and multi-scale characterization of biodegradable biocomposites fabricated via thermo-extrusion and thermo-compression. Three biodegradable biopolymers PLA, PBS, and PBAT were selected based on their projected development potential as matrices for sustainable packaging. Both agro-industrial by-products, Brewer’s Spent Grain and Orange Peel, were incorporated in their raw powdered form at 10 wt.%, without additional pre-treatment or compatibilizers, aligning with a low-carbon, circular economy approach.

A filler content of 10 wt.% was selected as a rational compromise between conflicting objectives. These included the following: (i) maximizing filler content to reduce polyester consumption; (ii) ensuring sufficient biodegradability under mesophilic conditions (20–45 °C), which will be assessed in Part B of this work; and (iii) maintaining acceptable processability and mechanical performance in the absence of any compatibilization strategy.

Indeed, in the absence of compatibilization strategies—such as filler pretreatment, matrix functionalization, or the use of coupling agents—raising the filler content beyond 10 wt.% would likely compromise mechanical and thermal performance. This effect would be particularly pronounced for tensile strength, as previously reported for similar agro-industrial by-products [42,43,44].

Therefore, this study focuses on characterizing the baseline properties—thermal, mechanical, and water sorption behavior—of uncompatibilized biocomposites, to provide a reference framework. This constitutes a critical first step toward the development of compatibilization strategies that are not only effective, but also economically viable, environmentally acceptable, and minimally detrimental to biodegradability. Such strategies may involve the pretreatment of the organic filler (e.g., acid or alkaline treatment), functional grafting of the matrix (e.g., maleic anhydride), surface modification of the filler (e.g., esterification or acetylation), the use of coupling agents, or copolymerization with other polymers.

This choice also reflects a deliberate low-input eco-design strategy [45], aimed at reducing the environmental footprint of biocomposite fabrication. It further seeks to preserve the inherent biodegradability of cellulosic fillers. This property is often diminished by non-biodegradable or toxic compatibilizers, such as silanes or isocyanates [45,46].

Thus, this study investigates the intrinsic effect of raw filler type as a function of the polyester matrix, independently of any chemical compatibilization. A full factorial design enables a systematic assessment of the interactions between three biopolymer matrices (PLA, PBS, and PBAT) and two agro-industrial fillers (OP and BSG), establishing a solid baseline for the future development of compatibilized systems [47].

All manufacturing parameters were kept constant, including organic filler proportion (wt.%), filler moisture content, the two-step fabrication process for biocomposites, screw rotation speed during extrusion, pressing time and pressure, and the pre-drying of the polyester matrix. The only variations concerned extrusion and pressing temperatures, adjusted between PLA-based and PBS/PBAT-based composites due to their distinct melting temperatures. Pure polyester films were produced in a single step by thermo-pressing.

This study aims to compare the effects of OP and BSG fillers in PLA, PBS, and PBAT matrices in terms of intrinsic properties—mechanical, thermal, and water vapor sorption behavior—with a particular focus, using modeling approaches (Park model), on the water vapor sorption kinetics of the materials.

*Part A* is exclusively devoted to the study of these intrinsic properties for the produced biocomposites, and to their comparison with pure polyester fragments manufactured under analogous conditions.

*Part B* will address the evaluation of the extrinsic capacity of these materials to biodegrade under decentralized composting conditions (i.e., domestic and community-scale), following an adapted version of the home-compostability specification standard NF T51-800 and the ISO 14855 protocol [48,49]. This will include the following: (i) quantifying the mineralization of these materials through the aerobic metabolic activity of microorganisms present in the decomposition medium, (ii) monitoring the evolution of structural properties during the process, (iii) isolating and identifying bacterial strains effective in polyester biodegradation through selective enrichment in minimal medium, and (iv) assessing the phytotoxicity of the resulting compost.

## 2. Materials and Methods

### 2.1. Raw Materials

#### 2.1.1. Agro-Industrial By-Products

Orange peel (OP)—Orange peels were collected from a waste compartment within an orange pressing unit made available to customers (versatile Pro 1 STEP, Zumex Group S.A., Valencia, Spain). The excess pulp was removed using a cutter to retain only the mesocarp and epicarp. This separation step aimed to eliminate pulp mainly composed of soluble compounds such as simple sugars, organic acids, and pectins [50], whose presence could lead to a sticky texture and hinder the homogeneity of the blend with the polymer matrix during single-screw thermo-extrusion. In contrast, dried and ground peel yields a powder richer in cellulose and hemicelluloses, which is more stable, cohesive, and better suited to thermoplastic processing requirements. In citrus processing, pulp and peel are commonly separated using centrifugal depulpers or screw/sieve-based systems [51], enabling distinct valorization routes for each fraction, such as anaerobic digestion or animal feed in the case of sugar-rich pulp. The peels were then dried in an oven at 60 °C until their weight stabilized.

Brewer’s spent grain (BSG)—Brewer’s spent grains were obtained from a local craft brewery (Brasserie de Sutter, Gisors, France). They originated from multiple brews using a malt blend composed on average of 85 wt.% barley and 15 wt.% wheat, primarily for blond beer production (90 vol.%) and, to a lesser extent, dark beer (10 vol.%). During the brewing process, the mash was filtered using a coarse 1 mm mesh sieve. The grains were then dried in an oven at 60 °C until their weight stabilized.

Grinding process—Both dried OP and BSG underwent two successive milling steps. The first step consisted of size reduction using a knife mill (Retsch GmbH, Haan, Germany) equipped with a 500 µm sieve, followed by finer milling in a high-speed rotor mill (PULVERISETTE 14, Fritsch GmbH, Idar-Oberstein, Germany) equipped with a 250 µm sieve. The resulting powders were stored in airtight bags and kept in the dark at room temperature until use.

As detailed in Section 2.2, both fillers were later adjusted to a moisture content of 25% to ensure stable flow during the extrusion process.

#### 2.1.2. Polyesters

A commercial polybutylene succinate (PBS) (PBE 003; MFI: 4–6 g/10 min, 190 °C, 2.16 kg; density: 1.26 g/cm^3^), an amorphous polylactic acid (PLA) (PLE 005-A; MFI: 3 g/10 min, 190 °C, 2.16 kg; density: 1.24 g/cm^3^), with a D-lactide content of 4% and an estimated melting point of approximately 160 °C, and a commercial poly(butylene adipate-co-terephthalate) (PBAT) (PBE 006; MFI: 4–6 g/10 min, 190 °C, 2.16 kg; density: 1.26 g/cm^3^), were supplied in granule form by Natureplast (Mondeville 14120, France) (Table 1). These polymers were used as the matrix materials for the fabrication of the composites investigated in this study.

### 2.2. Composite Preparation

The polyester granules (PLA, PBAT, and PBS) were used as supplied by the manufacturer but were pre-dried in an oven at 60 °C overnight prior to processing to remove residual moisture. The agro-industrial by-products used as fillers—brewer’s spent grain (BSG) and orange peel (OP)—were adjusted to a moisture content of approximately 25% (*w*/*w*), measured with an infrared moisture analyzer (SARTORIUS, MA35), which was found optimal in preliminary trials to ensure continuous and stable flow during extrusion.

The fabrication of the biocomposites (polymer/organic filler: 90/10; *w*/*w*) involved two sequential steps: (i) thermo-extrusion using a single-screw extruder (L/D = 24) (SCAMEX) to produce biocomposite granules, followed by (ii) thermo-compression using a thermo-press (15 T press, SCAMEX, Villeurbanne, France) to form thin films.

#### 2.2.1. Extrusion and Pellet Preparation

For PBS and PBAT, the extrusion temperature profile was set at 125 °C/130 °C/125 °C, whereas for PLA, the profile was 155 °C/155 °C/150 °C. The screw speed was maintained constant at 20 rpm throughout the extrusion process. The extruded filament was immediately cooled in a water bath at room temperature (~20 °C), with a short residence time in the bath (<10 s), before being granulated using a knife granulator (SCAMEX). The biocomposite granules were subsequently placed in a ventilated oven at 60 °C for 48 h to eliminate any residual moisture.

Mass flow rate tests were carried out under the same conditions as those of the single-screw extrusion step used for biocomposite fabrication, maintaining an identical temperature profile and screw rotation speed. Measurements were conducted on both neat polymers and biocomposite blends, in duplicate. Once the flow through the spaghetti dies visually stabilized, the extrudate was collected for exactly one minute and immediately weighed to determine the mass flow rate (g/min). These tests aimed to compare the flow performance of the different formulations with one another and relative to their corresponding neat polymer.

#### 2.2.2. Thermo-Compression and Film Fabrication

Prior to the thermo-compression step, both the biocomposite and neat polyester granules were dried again in an oven at 60 °C overnight. A total of 15 g of material was distributed between two sheets of unbleached parchment paper and then pressed for 90 s using the hydraulic closing pressure of the press. Subsequently, an additional pressure of 200 bars was applied for a further 90 s to reduce the sheet thickness. The applied plate temperatures were 135 °C for PBS and PBAT, and 165 °C for PLA.

**Figure 1:**
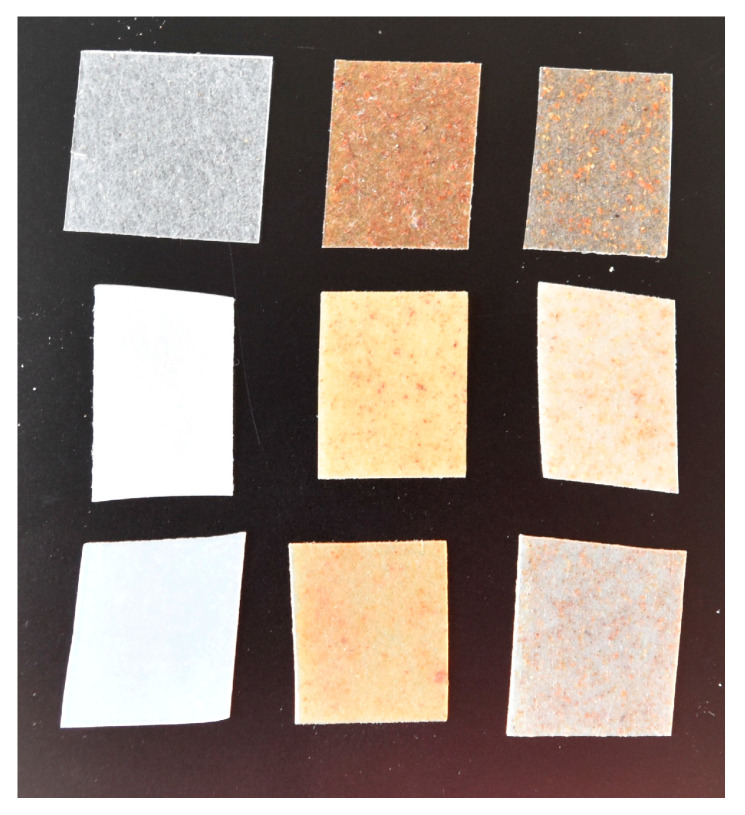
Photograph of materials fabricated by thermopressing. From left to right: pure polymer, biocomposite with orange peel (OP), biocomposite with brewer’s spent grain. From top to bottom: PLA and PLA-based composites; PBS and PBS-based biocomposites; PBAT and PBAT-based biocomposites.

Immediately after pressing, the polyester and biocomposite sheets were cooled to room temperature, stored in airtight plastic bags, and kept in the dark. The plastic sheets were then cut into square fragments (~2 cm × 2 cm) using a paper cutter. The average thickness of the fragments was determined by calculating the arithmetic mean of fifty measurements taken from different fragments using a digital caliper for each material. The thickness of the films was varied between 192 and 384 µm (Table 6). Figure 1 shows images of the biocomposite films obtained from thermo-compression process.

### 2.3. Characterization of By-Products and Materials

#### 2.3.1. Biochemical Fractionation Using the Van Soest Method

The relative contents of water-soluble compounds, cellulose, hemicellulose, and lignin/cutin in the agro-industrial by-products were estimated using 1 g of finely ground dried material, sieved through a 1 mm mesh, following the biochemical fractionation method of Van Soest [52].

Fiber analysis was performed in triplicate using the Fibretec™ 8000 (Foss Analytical A/S, Hillerød, Denmark) system with porous glass crucibles. The samples were sequentially exposed to different solutions to selectively solubilize their constituents. Initially, they were treated with a neutral detergent fiber (NDF) solution at 100 °C for one hour, rinsed with acetone, dried overnight at 105 °C, and weighed. The same protocol was then applied using an acid detergent fiber (ADF) solution, followed by a final hydrolysis step with 72% (*v*/*v*) sulfuric acid (ADL). These treatments allowed for the isolation of different fractions: the mass loss after NDF treatment corresponded to the water-soluble compounds, the difference between NDF and ADF represented the hemicellulose content, the difference between ADF and ADL corresponded to the cellulose content, while the final residue after ADL treatment consisted of the most stable compounds, primarily lignin and cutin.

#### 2.3.2. Determination of Molecular Mass and Polydispersity Index

The number-average molecular weight (Mn) (PMMA equivalent), mass-average molecular mass (Mw) (PMMA equivalent), and polydispersity index (PDI = Mw/Mn) of the materials were determined using size-exclusion chromatography (SEC). Prior to analysis, the samples were solubilized overnight at room temperature in hexafluoroisopropanol (HFIP) until the complete dissolution of the polymers. Biocomposite fragments were subsequently filtered (0.2 µm) to remove undissolved organic filler particles.

An ultra-high-performance liquid chromatography (UHPLC) system (Waters APC Acquity system, Waters Corporation, Milford, MA, USA ) was used, consisting of a pre-column (Acquity APC XT450 2.5 µm 4.6 × 30 mm), three columns (Acquity APC XT450 2.5 µm 4.6 × 75 mm, Acquity APC XT125 2.5 µm 4.6 × 75 mm, Acquity APC XT45 1.7 µm 4.6 × 150 mm) maintained at 35 °C, and a refractive index (RI) detector. Results were obtained based on a conventional PMMA calibration (RI at 35 °C).

#### 2.3.3. Determination of Specific Surface Area Using the BET Method

The BET specific surface areas of the two agro-industrial by-products were determined by nitrogen physisorption at 77 K using a micro porosimeter (Gemini 2390t; Micromeritics). Prior to analysis, the samples, previously dried at 50 °C, were degassed under vacuum (<0.05 mbar) for 24 h at the same temperature using a vacuum preparation system (VacPrep 061; Micromeritics) to remove residual water and volatile organic molecules that could interfere with the measurements.

The BET equation (Equation (1)) expresses the relationship between the total amount of gas molecules adsorbed (X) and a specific relative pressure (*P/P_0_*), with *C* representing a secondary parameter associated with the heat of adsorption, and X_m_ representing the amount of gas adsorbed to form a monolayer [53]:

(1) $$\frac{1}{Χ\;[{(P}_{0}/P)\;-\;1]}\;=\;\frac{1}{{Χ}_{m}∁}+\frac{∁\;-\;1}{{Χ}_{m}∁}\;\left(\frac{P}{P_{0}}\right)$$

The linear form of this equation (Equation (1), right-hand side) allows for the determination of the parameters from which the specific surface area of the sample is calculated according to Equation (2)

(2) $$SA=\frac{1}{slope+intercept}\;CSA$$

where *CSA* is the cross-sectional area of the adsorbate [54].

The BET surface areas were calculated from experimental data obtained during the sorption stage over a relative pressure range (*P/P_0_*) from 0.05 to 0.30.

#### 2.3.4. Particle Size Distribution of By-Product Particles

The particle size distribution (0.01 µm to 10,000 µm) of the by-product particles used as fillers was estimated using a laser granulometer equipped with a solid matrix detection module (Mastersizer—Sirocco 2000; United Kingdom, Malvern Panalytical Ltd., Malvern, Worcestershire, UK). Three parameters were identified to characterize the particle size distribution of the samples and allow for comparisons. The d10 parameter represents the particle diameter below which 10% of the particles fall. Similarly, d50 represents the median particle size, and d90 corresponds to the particle diameter at which 90% of the cumulative distribution is reached.

#### 2.3.5. Skeletal Density

The skeletal density of the materials (neat polymers and biocomposites) and of the agro-industrial by-products was determined using a helium pycnometer (Pycnomatic-ATC, Porotec GmbH, Hofheim, Germany). Three replicates were performed until the relative standard deviation ($CV(\%)=\frac{\sigma }{\mu }\cdot 100$) between the replicates was below 0.5%.

#### 2.3.6. Scanning Electron Microscopy (SEM)

The surface and cross-section of the biocomposite samples cryo-fractured in liquid nitrogen were examined using scanning electron microscopy (SM–5000 Neoscope™, JEOL Ltd., Tokyo, Japan), operated at an accelerating voltage of 10 kV in secondary electron detection mode (SED). Prior to observation, the samples were coated with a thin layer of gold to ensure electrical conductivity. These analyses aimed to provide visual information on the integration of the organic filler within the polymer matrix. The organic fillers alone were also examined under the same experimental conditions.

#### 2.3.7. Fourier Transform Infrared Spectroscopy (FT-IR)

The by-products and the processed plastic fragments (biocomposites and neat polyesters) were characterized by Fourier Transform Infrared Spectroscopy (FTIR) using a spectrometer (Nicolet iS10, Thermo Scientific, Waltham, MA, USA) with an attenuated total reflectance (ATR) acquisition technique (ZnSe—ATR) (Smart iTX™ ATR, Thermo Scientific, Waltham, MA, USA).

Each sample was subjected to 32 scans at a resolution of 4 cm^−1^. Background noise was systematically recorded before each measurement. Four replicates were performed on different fragments to establish an average IR spectrum, calculated as the arithmetic mean of all individual spectra. The acquired spectra were analyzed using the OMNIC 8.1 software. The average spectrum of each formulation underwent numerical ATR correction, smoothing, and manual baseline correction.

#### 2.3.8. Thermal Stability Study by Thermogravimetric Analysis (TGA)

Thermogravimetric analysis was conducted in triplicate for each material sample, which had been pre-dried for 24 h at 40 °C in an oven (20 ± 2 mg per Al_2_O_3_ crucible), using a thermobalance (TG 209 F1 Libra, NETZSCH-Gerätebau GmbH, Selb, Germany). The measurements were performed under a non-oxidizing gas atmosphere (argon, 10 mL/min) over a temperature range of 20–600 °C at a heating rate of 10 K/min.

The generated thermograms were analyzed using the Proteus Analysis software, version 9.0 for TGA (NETZSCH-Gerätebau GmbH, Selb, Germany). Two parameters were extracted from the thermograms: T_d5%,_ the temperature at which the sample exhibited a 5% weight loss, and T_dpeak_, the temperature at which the thermal decomposition rate was highest.

#### 2.3.9. Differential Scanning Calorimetry (DSC) Measurements

Heat capacity measurements of the materials were performed in triplicate using differential scanning calorimetry (DSC 214 Polyma, NETZSCH-Gerätebau GmbH, Selb, Germany). Between 8 mg and 12 mg of each sample was subjected to a series of heating and cooling cycles.

For PBS and PBAT, the applied program consisted of an initial heating cycle from ambient temperature up to 200 °C, followed by a 3 min isothermal phase at 200 °C, then cooling down to −65 °C to eliminate thermal history effects. A second heating cycle was then performed from −65 °C to 200 °C. For PLA, the maximum temperature reached in the first cycle was 180 °C, followed by cooling to 0 °C and a subsequent reheating to 180 °C. For the first and second cycles, the heating and cooling rates were set at 10 °C/min. The carrier gas used was argon, with a flow rate of 40 mL/min.

The parameters of interest, the glass transition temperature (T_g_), the melting temperature (T_m_), the melting enthalpy (ΔH_m_) and the cold crystallization enthalpy (ΔH_cc_) were measured during the first and second heating cycles.

The crystallinity index of the samples was calculated using the following equation:

(3) $$ꭓ\;\left(\%\right)=\;\frac{\left({\Delta H}_{m}\;-\;{\Delta H}_{cc}\right)}{\;{\Delta H{}^{\circ}}_{m\;}\left(1-\emptyset \right)}\;\times \;100$$

where ΔH_m_ is the measured melting enthalpy, ΔH_cc_ is the measured cold crystallization enthalpy, ΔH°_m_ corresponds to the theoretical standard melting enthalpy of a fully crystalline material, and ϕ represents the estimated mass fraction of the fillers in the biocomposite (~7.5%).

The reference values for ΔH°_m_ used for each polymer were as follows: PLA (93 J/g), PBS (110.3 J/g), and PBAT (114 J/g) [55,56]. The DSC thermograms were analyzed using the Proteus Analysis software, version 8.0 for DSC (NETZSCH-Gerätebau GmbH, Selb, Allemagne).

Although the experimental uncertainty is lower than 5% for the crystallinity rate and 1 °C for the glass transition temperature (T_g_), minimum uncertainties of ±5% and ±1 °C, respectively, are applied to account for potential systematic errors.

#### 2.3.10. Tensile Strength Tests

Tensile strength tests were performed using a universal testing machine ( Autograph AGS-X, Shimadzu Corporation, Kyoto, Japan) equipped with a 200N load cell, following the ISO 527:3 (1B) standard [57], at a temperature of 23 ± 2 °C and a relative humidity of approximately 50%. The 1B-shaped specimens were obtained by machining with a laser cutter (MLLASER, ML-W960).

The average thickness of each specimen was determined as the arithmetic means of four independent measurements taken with a digital caliper at the narrowing section of each sample. These independent thickness measurements were entered into the Trapezium, version X software (Shimadzu Corporation, Kyoto, Japon) interface to normalize the tensile strength results. The calculated parameters were as follows:

- The Young’s modulus E (MPa) at a test speed of 1 mm/min
- The tensile strength σ (MPa) and elongation at break ε (%), measured at a test speed of 50 mm/min (for PBAT and PBS) and 5 mm/min (for PLA).

Six samples were tested for each material/parameter pair to compute an average value. A one-way analysis of variance (ANOVA) was performed separately for each polymer-property combination. When a significant difference was detected (α = 0.05), Tukey’s HSD post hoc multiple comparison test was applied to identify statistically significant differences (α = 0.05) within the groups.

#### 2.3.11. Contact Angle Measurements and Surface Energy Determination

Static contact angle measurements were conducted on film samples using the drop shape analyze (DSA25—Krüss Germany) at room temperature (22 ± 1 °C) using the sessile drop method. Three different liquids (ultrapure deionized water, diiodomethane, and ethylene glycol) were tested to calculate the surface energy of the samples and their respective polar and dispersive components.

A 5 µL drop of each liquid was deposited on the sample surface using a micro-syringe. The contact angle formed at the interface between the drop and the polymer membrane was measured using ADVANCE software (Krüss GmbH, Hambourg, Allemagne), based on images captured by a CCD camera.

For each liquid-sample pair, the contact angle value was calculated as the arithmetic mean of 15 measurements performed on three different fragments (five measurements per fragment) to minimize experimental errors. The surface energy was then determined using the Owens–Wendt–Rabel–Kaelble (OWRK) model:

(4) $$\gamma_{sl\;}=\gamma_{s}+\gamma_{l}\;-2\;({\left(\gamma_{s}^{d}\gamma_{l}^{d}\right)}^{1/2}+{\left(\gamma_{s}^{p}\gamma_{l}^{p}\right)}^{1/2})$$

where *γ_sl_* is the interfacial tension between the solid and liquid, *γ_S_* is the surface energy of the solid in the presence of the liquid vapor, and *γ_L_* is the surface tension of the liquid.

By combining this equation with the Young and Dupré equations [58,59], it can be rewritten as

(5) $$\gamma_{l}\;(1+cos\;\theta )=2\;({\left(\gamma_{s}^{d}\gamma_{l}^{d}\right)}^{1/2}+{\left(\gamma_{s}^{p}\gamma_{l}^{p}\right)}^{1/2})$$

where θ is the contact angle at the interface of the three phases. Since the polar and dispersive components of the applied liquids are known a priori, the two unknowns, ($\gamma_{s}^{d}\;and\;\gamma_{s}^{p})$, can be determined using the least squares method. Finally, the total surface energy of the samples was calculated as follows:

(6) $$\gamma_{s}=\;\gamma_{s}^{d}+\gamma_{s}^{p}$$

#### 2.3.12. Dynamic Water Vapor Sorption (DVS)

The water vapor sorption capacity of the samples was measured at 28 °C using a microbalance under controlled humidity (SPSx-1µ High Load, ProUmid GmbH & Co. KG, Ulm, Germany). The samples were pre-dried at 40 °C in an oven for 24 h before being placed (~200 mg) into the measurement system. The relative humidity (RH) was initially set to 0% until the sample weight stabilized (dm/dt = 0.01%) with a stabilization time of 15 min.

Humidity levels were then incremented in 10% steps from 0% to 90%, followed by a desorption sequence from 90% to 0%, allowing for the measurement of water sorption and desorption isotherms over this humidity range. The water concentrations taken at the equilibrium state (C_eq_) of each sorption kinetic as the function of water activity (*a*) allow to plot the isotherm curve C_eq_ = f(a).

#### 2.3.13. Modeling of Sorption Isotherms (Park Model)

The water sorption isotherms at 28 °C obtained by Dynamic Vapor Sorption (DVS) were modeled using the multimodal sorption model developed by Park [60], which is widely recognized and frequently applied to describe the water vapor sorption behavior of bio-based polyesters and cellulose-based compounds. Unlike other models such as GAB, the Park model simultaneously accounts for Henry, Langmuir, and clustering sorption modes, making it well suited to the sigmoidal isotherms and multi-mechanistic water uptake observed in our semi-crystalline polyesters and biocomposites.

This model is divided into three different contributions, each corresponding to a specific sorption mechanism: (i) for low water activity (*a* ≤ 0.1), the Langmuir model describes the adsorption of water molecules onto specific sites (polar functional groups and microvoids) on the sample surface; (ii) for intermediate water activity (0.2 ≤ *a* ≤ 0.5), Henry-type sorption is predominant; (iii) for high water activity (*a* > 0.5), the sorption kinetics follow an exponential behavior, reflecting the aggregation of water molecules within the material.

The model can be expressed as follows:

(7) $$C_{eq}=\frac{A_{L}b_{L}a_{w}}{1+b_{L}a_{w}}+\;k_{h}a_{w}+nK_{a}k_{h}^{n}a_{w}^{n}$$

where *A_L_* is the Langmuir site concentration, *b_L_* is the affinity constant of water molecules for Langmuir sites, *k_h_* is Henry’s law constant, *K_a_* is the water molecule aggregation constant, and *n* represents the average number of molecules per aggregate. Since *C_eq_* is expressed in grams of water per gram of dry material, all coefficients are dimensionless.

To assess the accuracy of the Park model in describing the experimental water sorption isotherms of various materials, the Mean Relative Deviation (*MRD*) was calculated using the following equation:

(8) $$MRD\;(\%)\;=\;\frac{100}{N}\sum_{i\;=\;1}^{N}\frac{\left|m_{i}-m_{pi}\right|}{m_{i}}$$

where *m_i_* is the experimental value, m*_pi_* is the predicted value from the model, and *N* is the number of experimental data points. A deviation module value below 10% is commonly considered indicative of a good agreement between experimental and predicted values [61].

The curves of the five-parameter Park model were fitted by non-linear regression using the Excel Solver (Microsoft Excel for Microsoft 365—Version 2401), minimizing the sum of squared residuals between the model predictions and the experimental data.

#### 2.3.14. Water Diffusivity

Two half-sorption coefficients (D_1_ and D_2_), representing the rate of water diffusion within the material normalized by its thickness, were calculated from water sorption isotherms measured by DVS for a given water activity. D_1_ corresponds to the diffusion rate during the first half of the sorption process, when M (t)/M_eq_ < 0.5, whereas D_2_ represents the diffusion rate during the second half, when M (t)/M_eq_ > 0.5. Assuming a constant diffusion coefficient, the progression of the sorption process ($M(t)/M_{eq})$ as a function of time can be determined by solving Fick’s law:

(9) $$\frac{M(t)}{M_{eq}}=\frac{4}{\surd \pi }\surd \tau \left[1+2\surd \pi \sum_{n=1}^{\infty }{\left(-1\right)}^{n}\;ierf\frac{n}{2\surd \tau }\right]\;=\;1-\frac{8}{\pi^{2}}\sum_{n=0}^{\infty }\frac{e^{-{\left(2n+1\right)}^{2}\pi^{2}\tau }}{{\left(2n+1\right)}^{2}}$$

Solving Equation (9) allows for the determination of two distinct diffusion coefficients:

D_1_, applicable for short time periods (($M(t)/M_{eq})$ < 0.5), is calculated using

(10) $$\frac{M(t)}{M_{eq}}\;\approx \;\frac{4}{\surd \pi }\surd \tau \;\approx \frac{4}{L}\sqrt{\frac{D_{1}}{\pi }}\surd t$$

D_2_, corresponding to longer durations (($M(t)/M_{eq})$ > 0.5), is given by

(11) $$ln\left[1-\frac{M(t)}{M_{eq}}\right]\;\approx -\pi^{2}\tau -ln\left[\frac{\pi^{2}}{8}\right]\;\approx -\pi^{2}\frac{D_{2}t}{L^{2}}-ln\left(\frac{\pi^{2}}{8}\right)$$

All calculations were performed using Microsoft Excel software.

#### 2.3.15. Mean Cluster Size (*MCS*)

The mean cluster size of water molecules formed within the materials was estimated using the Zimm and Lundberg [62] method, and is defined as follows:

(12) $$MCS\;=\;1\;+\;\left[\frac{\Phi_{s}G_{s}}{V_{s}}\right]$$

where $G_{s}$ represents the integral aggregation, $V_{s}$ is the molar volume of the penetrant, and *α* and $\Phi_{s}$ are the activity and volume fraction of the penetrant, respectively.

According to Starkweather [6], this expression can be rewritten as

(13) $$MCS=(1-\Phi_{s}){\left[\frac{\partial \;ln\;\Phi_{s}}{\partial \;ln\;a}\right]}_{p,T}$$

When applied to the Park model, the *MCS* becomes

(14) $${MCS}_{Park}=\frac{p^{2}}{{M^{3}(1+p/M)}^{2}}\left[k_{H}.a+\frac{A_{l}.b_{l}.a_{w}}{{\left(1+b_{l}.a_{w}\right)}^{2}}+K_{a}.n.a_{w}^{n}\right]$$

where *p* is the ratio of the apparent density of water to the apparent density of the material $\left(p_{w}/p_{p}\right)$, and *M* is the equilibrium mass gain.

From a physical standpoint, the *MCS* parameter provides an estimate of the average number of water molecules aggregated into clusters within the material at a given water activity. Higher values indicate stronger water–water interactions and more extensive clustering, whereas lower values suggest a predominance of water–material interactions.

All *MCS* calculations were performed using Microsoft Excel software.

## 3. Results and Discussion

### 3.1. Fabrication of Biocomposites

The biocomposite materials were successfully fabricated under the defined operating conditions, following a two-step process: (i) blending of the filler and polymer matrix by thermo-extrusion, and (ii) shaping by thermo-compression (Figure 1).

Although twin-screw extrusion is generally recommended for biocomposite formulations with high lignocellulosic filler contents (≥20%), a single-screw extruder was employed in this study for both practical and technical reasons. The equipment used reflects a low-energy, small-scale processing configuration, in line with the eco-design objectives of this work. Furthermore, several studies have shown that single-screw extrusion can achieve a satisfactory dispersion of agro-industrial fillers in PLA or PBS matrices at similar loadings (5–15 wt.%) without significantly compromising material performance [43,63,64,65].

Nevertheless, the extrusion of biocomposites, particularly those containing brewer’s spent grain (BSG), was highly sensitive to the moisture content of the filler. Below 20% (*w*/*w*), bulk flow behavior deteriorated markedly, likely due to increased interparticle friction and/or mechanical drag against the metallic walls of the extruder, which impeded the formation of a continuous strand from the polyester–BSG blend. Incremental trials, increasing the moisture content from 5% to 30% in 5% intervals, identified 25% as the optimal level, with water presumably acting as a plasticizer and/or lubricant [66]. This moisture level was subsequently applied to both fillers (BSG and OP) to ensure consistent processing conditions across all biocomposite formulations.

Although this approach—processing with lignocellulosic fillers at non-zero moisture levels—is less common in standard industrial practice due to the risk of hydrolytic degradation, several studies have shown that it remains technically feasible for biodegradable polyesters. For instance, Berthet et al. [67] examined PHBV-based biocomposites incorporating wheat straw fibers with moisture contents varying from ~2 wt.% to ~15 wt.% and reported a decrease in the polymer weight-average molecular weight (M_w_) from 242,376 g/mol (for dry fibers) to 201,046 g/mol (for humid fibers), i.e., −17%. This reduction was described as moderate in their study and demonstrates that processing under controlled conditions with moderate moist fillers can remain compatible with thermoplastic biopolymer extrusion.

Additionally, residual filler moisture may induce a moderate reduction in molecular weight through chemical hydrolysis, which can enhance polymer matrix biodegradability under mesophilic conditions [68], supporting the complete elimination of these materials after use.

Moreover, fully drying agro-industrial fillers involves substantial energy consumption [69], which should ideally be avoided—or at least minimized—within a low-carbon bioplastic production framework. This further underscores the relevance of using partially moist fillers, provided that process stability and final material performance are not significantly compromised. For instance, the drying of wet lignocellulosic materials such as paper pulp has been estimated to require between 2.8 and 4.0 gigajoules per ton of evaporated water [70]. However, the actual energy demand can vary considerably depending on the drying technology employed.

The mass flow rate results obtained under real extrusion conditions are presented in Table 2. The incorporation of organic fillers led to a significant decrease in the mass flow rate of the blends. The incorporation of lignocellulosic fillers in micro-composites (particle size > 1 µm) generally leads to a reduction in mass flow rate due to increased resistance to flow within the molten polymer matrix. This phenomenon is mainly attributed to the solid particles acting as physical obstacles, disrupting polymer chain mobility, promoting interparticle friction, and thereby increasing the melt viscosity [71]. A more pronounced reduction was observed with orange peel (OP) compared to brewer’s spent grain (BSG). This could be explained, at least in part, by the high protein content of BSG, which tends to lower the melt viscosity and facilitate flow through the extruder, in contrast to other cellulosic fillers. For example, Hejna et al. [72] reported a melt flow rate nearly twice as high for PCL/BSG biocomposites compared to PCL/wheat bran blends, attributing this effect to the higher content of free amino acids in BSG (21 wt.%) versus wheat bran (4 wt.%).

### 3.2. Chemical Composition of Agro-Industrial By-Products

The main physicochemical characteristics of the agro-industrial by-products are summarized in Table 3. The biochemical fractionation analysis of these by-products indicates that brewers’ spent grain (BSG) is more fibrous than orange peel (OP) (Cellulose: OP 15.3 ± 0.3%; BSG 49 ± 0.5%) (Hemicellulose: OP 3.9 ± 0.4%; BSG 24.6 ± 0.4%). Given its markedly higher cellulose content, BSG is expected to induce greater rigidification in the resulting biocomposites compared to OP. Orange peel mainly consists of water-soluble constituents (WPC: OP 80.1 ± 0.2%; BSG 14.4 ± 0.1%). In the case of OP, these soluble compounds can be primarily attributed to the presence of simple carbohydrates (i.e., glucose, fructose, galactose, arabinose, and galacturonic acid) [73]. The total nitrogen content of brewers’ spent grain (3.4%, w) is 3.8 times higher than that of orange peel. This higher nitrogen content is associated with its significant concentration of non-solubilized proteins remaining after the brewing process, mainly found in the grain husk, pericarp, and hull [74].

### 3.3. BET Specific Surface Area and Particle Size Distribution of Ground Agro-Industrial By-Products

**Figure 2:**
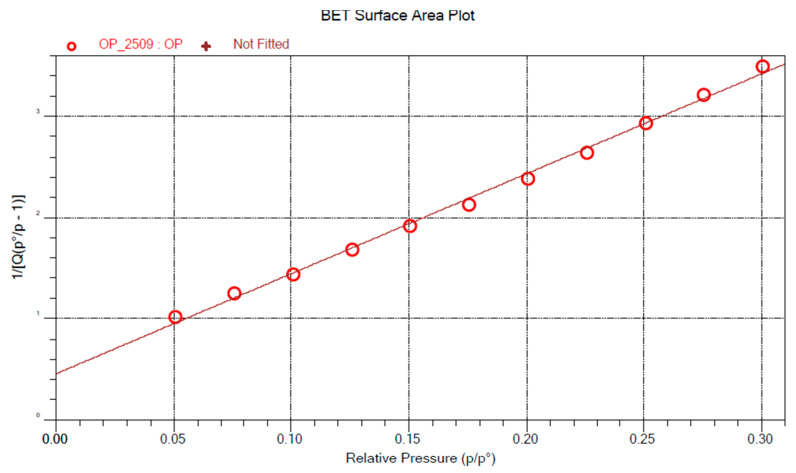
t-plot of N_2_ physisorption measurements of an orange peel (OP) sample according to the BET method during an adsorption cycle in the relative pressure range (P/P0) 0.05–0.30.

**Figure 3:**
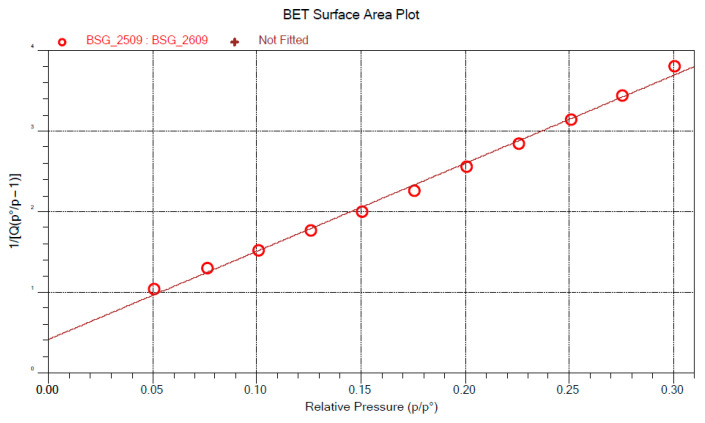
t-plot of N_2_ physisorption measurements of a brewer’s spent grain (BSG) sample according to the BET method during an adsorption cycle in the relative pressure range (P/P0) 0.05–0.30.

The determination of the BET specific surface area is based on the application of the Brunauer, Emmett, and Teller (BET) theory, which describes the physical adsorption of gas molecules onto a solid surface. Gas molecules can penetrate between particles, into open pores, cracks, and surface textures, allowing the calculation of the microscopic surface area per unit mass of the samples [75]. The linearization of the BET model equation enables the application of a linear regression model to verify the validity range of the model. The correlation coefficients (BSG: 0.998; OP: 0.998) calculated from the data for both by-products indicate that the model is reliable within the range of relative pressures used during the sorption cycle. Therefore, the BET model parameters can be used for further analysis (Figure 2 and Figure 3).

The BET specific surface area of orange peel particles (0.4207 ± 8.10^−3^ m^2^/g) is higher than that of brewers’ spent grain (0.3838 ± 8.10^−3^ m^2^/g). The BET surface area is a key indicator of the potential reactivity of the filler and its interaction with the polymer matrix. A higher specific surface area suggests that the filler particles establish a better interface within the biocomposite, which may enhance stress transfer [76]. The BET surface values obtained for orange peel particles are consistent with those reported by Praipipat et al. [77], who measured a surface area of 0.431 m^2^/g.

**Figure 4:**
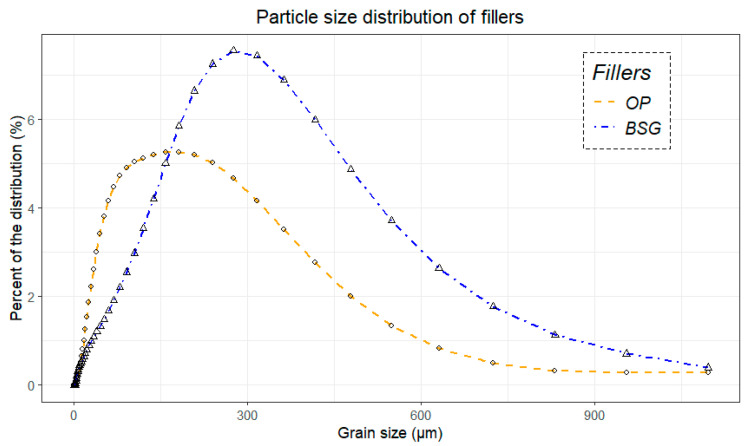
Particle size distribution of filler particles (OP and BSG) estimated using laser granulometry equipped with a detection module for solid matrices. Triangles and squares represent the experimental data points, while the lines correspond to regression curves fitted to the experimental data.

The particle size distribution curves of both agro-industrial by-products, determined by laser diffraction, are presented in Figure 4. The average particle volume, estimated using laser diffraction, is 165.35 µm for orange peel (obscuration rate: 5.06) and 245.40 µm for brewers’ spent grain (obscuration rate: 2.19). The distribution is also more uniform (0.98) for orange peel, with particle sizes concentrated at smaller values (d_10%_: 27.78 µm; d_50%_: 112.03 µm; d_90%_: 349.23 µm) compared to brewers’ spent grain (0.66) (d_10%_: 39.22 µm; d_50%_: 210.95 µm; d_90%_: 486.55 µm).

The presence of particles exceeding the mesh size (250 µm) used during sieving can be attributed to several factors. First, laser diffraction measures an equivalent spherical volume, which does not necessarily reflect the actual dimensions of the particles—especially when they are fibrous or irregular in shape, as observed for brewers’ spent grain (Figure 10). As a result, elongated or flattened particles may pass through a 250 µm sieve despite exceeding this size in other axes.

Second, although the fillers were dried prior to analysis, weakly bound residual moisture may remain within the particles and promote the formation of particle agglomerates [78]. Finally, electrostatic forces can also induce aggregation among surface-charged particles, particularly for lightweight materials with a high specific surface area [79].

Regarding biocomposites, the extent of particle aggregation is primarily governed by the balance between attractive and repulsive forces acting between the particles. Key factors influencing this phenomenon include surface tension, particle size distribution, morphology, and the shear forces applied during component mixing [80]. Finer filler particles generally exhibit a more homogeneous distribution within the biocomposite, whereas larger particles tend to aggregate due to gravitational and hydrodynamic attractive forces. A smaller particle size (i.e., ≤200 µm) is generally associated with the production of more stable composites [81].

In summary, orange peel particles exhibit a higher specific surface area, smaller average particle size, and greater homogeneity, all of which contribute to improved wettability with the polymer matrix. In contrast, brewers’ spent grain particles are larger, more heterogeneous, and possess a lower specific surface area, which likely promotes the formation of aggregation zones and weak points within the material structure.

### 3.4. Molecular Mass

The mechanical, thermal, and biodegradability properties of thermoplastic materials are closely linked to the molecular weight of the polymers and their distribution [82,83,84]. To investigate this, the number-average (M_n_) and mass-average (M_w_) molecular mass (PMMA equivalent) and the polydispersity index (PDI = M_w_/M_n_) of the polymer matrices in the plastic samples were determined using SEC after the material manufacturing process. These values are reported in Table 4.

**Figure 5:**
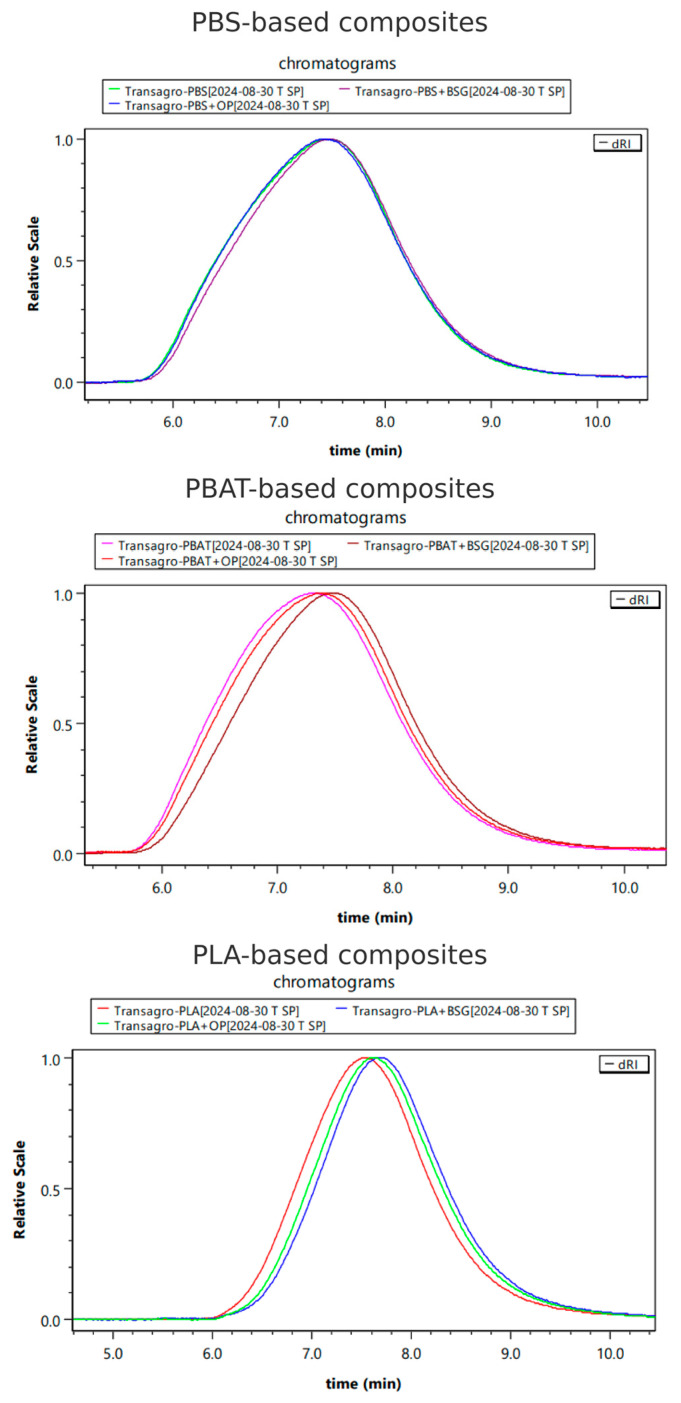
HFIP SEC chromatograms of the materials (RI signal).

Molecular mass distributions exhibit a unimodal pattern across all materials (Figure 5). A decrease in mass-average molecular mass (M_w_) is observed for all biocomposites compared to neat polymers, ranging from −2.52% to −22.02%, depending on the sample. Brewers’ spent grain-based biocomposites consistently show a greater reduction in M_w_ compared to orange peel-based biocomposites. PLA-based biocomposites also exhibit a more pronounced reduction in molecular weight compared to those based on PBS or PBAT.

However, the reduction in molecular weight remains moderate and within acceptable limits, even for PLA/BSG, which exhibited the most pronounced decrease (−22.02%). Its weight-average molecular mass remains above 70,000 g/mol, ensuring the retention of satisfactory thermal and mechanical properties of the polymer matrix.

Additionally, partial hydrolysis of polymer chains during processing, induced by residual filler moisture, can generate terminal –COOH groups. These may form hydrogen bonds or dipole–dipole interactions with the polar functional groups present in the agro-industrial filler, thereby enhancing matrix–filler compatibility.

PLA is a thermoplastic polymer known for its high sensitivity to processing conditions [85]. The molecular weight of polyesters—and PLA in particular—can decrease during melt processing, especially when thermo-extrusion is employed. This molecular degradation may result from the combined effects of elevated temperatures and mechanical shear stress and tends to increase with residence time within the extruder [86]. In addition, the chemical hydrolysis of macromolecular chains may occur in the presence of residual moisture, promoting ester bond cleavage and further reducing the polymer’s molar mass [87].

Polydispersity index values (molecular mass distribution) slightly decrease for biocomposite polymer matrices (Table 4), leading to a more homogeneous molar mass distribution alongside the decrease in M_n_ and M_w_. This suggests that the reduction in M_w_ is slightly more significant than that of M_n_, indicating that the underlying degradation mechanism is predominantly chain-end scission [88]. Thus, the observed preferential degradation pathway appears to result from the partial hydrolysis of the polymer chains, induced by the residual moisture present in the organic fillers. This phenomenon occurs because the polyester chain ends are more susceptible to chemical hydrolysis due to the presence of terminal –COOH and –OH groups, which contribute to local pH reduction and increased local solubility through hydrogen bonding with water [89].

The magnitude of the molecular weight reduction observed in PLA-based biocomposites is comparable to that reported by other authors for neat PLA processed under strictly controlled residual moisture conditions. This suggests that the processing itself, particularly the shear forces generated during extrusion and the elevated processing temperatures, as well as other manufacturing conditions such as compression or mixing intensity—may be sufficient to induce such a reduction.

For example, Carrasco et al. [90] investigated the influence of processing methods on the molecular weight of PLA. To minimize residual moisture and prevent hydrolysis during processing, PLA pellets were first dried under optimized conditions—4 h at 80 °C under reduced pressure. Two processing routes were then compared for their impact on the molecular weight of the initial PLA: (i) direct compression injection molding (100 bar, 180–210 °C), and (ii) single-screw thermo-extrusion (L/D = 25, 145–190 °C) followed by compression injection molding. The extruded PLA strand was cooled in a water bath at the die exit. It was observed that injection molding alone led to a 19.2% decrease in weight-average molecular weight (M_w_), whereas the extrusion followed by molding resulted in a 24.5% decrease.

Regarding the greater molecular weight reduction observed for brewer’s spent grain (BSG)-based biocomposites compared to those containing orange peel (OP), this trend is consistent across all studied matrices. A 22.02% decrease in weight-average molecular weight (M_w_) was measured for PLA/BSG formulations, versus 16.4% for PLA/OP. Similar trends were observed for the other matrices, with M_w_ reductions of 8.92% and 20.46% for PBS/BSG and PBAT/BSG, respectively, compared to only 2.52% and 6.82% for their OP-based counterparts.

Both weight-average (M_w_) and number-average (M_n_) molecular weights were lower than those of the neat polymers, indicating that degradation affects both long chains (M_w_) and short chains (M_n_). Furthermore, the polydispersity index (PI), calculated as the ratio M_w_/M_n_, showed a slight decrease for BSG biocomposites compared to OP formulations. For example, for PLA, the PI decreased from 2.08 in the neat polymer to 1.99 in PLA/BSG, whereas it was 2.05 for PLA/OP. This more pronounced reduction in polydispersity for BSG biocomposites suggests a narrower molecular weight distribution, potentially reflecting a predominance of chain-end scission rather than random scission, supporting the hypothesis of increased hydrolytic degradation in these formulations.

These results suggest that, despite identical moisture content and a slightly shorter residence time for BSG composites, specific interactions between the polymer matrix and the BSG filler promote enhanced hydrolysis, leading to a more pronounced decrease in the molecular weight of polymer chains compared to OP-based formulations.

### 3.5. FT-IR Analysis

**Figure 6:**
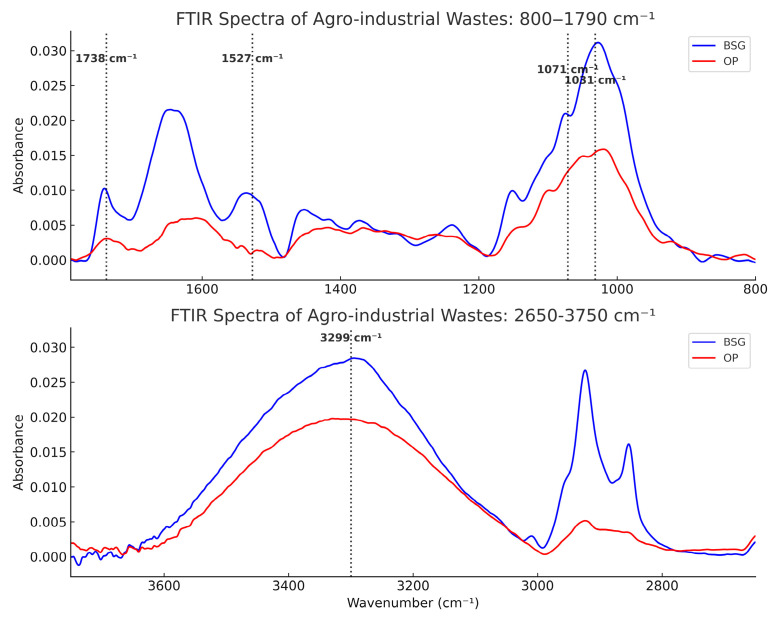
Zoomed-in FT-IR spectra of the two agro-industrial co-products (OP and BSG) in the regions 800–1790 cm^−1^ and 2650–3750 cm^−1^.

**Figure 7:**
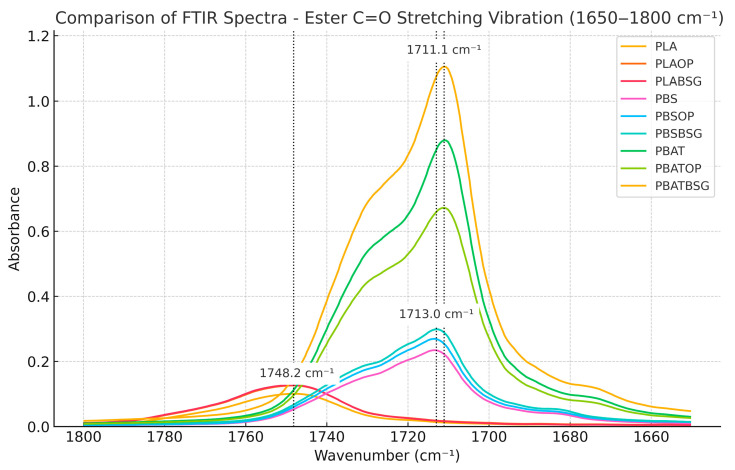
Zoomed-in FT-IR spectra of shaped materials (neat polymers and biocomposites) in the 1650–1800 cm^−1^ region, highlighting the –C=O ester stretching band.

The FT-IR spectra analysis reveals the presence of the main functional groups on the surface of the materials and agro-industrial by-products, associated with their characteristic absorption bands (Figure 6 and Figure 7).

For agro-industrial by-products, a broad band between 3000 cm^−1^ and 3600 cm^−1^ is observed. This band is typically associated with the stretching vibration of –OH and –N-H bonds, which are present in carbohydrates, organic fatty acids, and proteins. The bands located around 1071 cm^−1^ and 1032 cm^−1^ correspond to –C-O stretching vibrations and are characteristic of functional groups of cellulosic materials (glycosidic bond), due to the presence of hemicellulose and cellulose [73]. The peak at 1739 cm^−1^ can be attributed to acetyl-ester groups and uronic acid esters in hemicellulose, and to ester bonds of other phenolic compounds found in lignin and/or hemicellulose [91]. The peak at 1527 cm^−1^ is associated with –C=C bonds of aromatic rings present in lignin.

The relative intensities of these peaks (1032 cm^−1^, 1071 cm^−1^, 1739 cm^−1^, and 3290 cm^−1^) are higher for brewers’ spent grain (BSG), which aligns with the biochemical fractionation results, revealing a higher relative proportion of hemicellulose and cellulose in this by-product. The peak at 1527 cm^−1^ is exclusively present in the BSG spectrum, confirming the very low lignin content in orange peel (OP).

For polyesters and their composites (Figure 7), a characteristic stretching vibration band of the ester carbonyl (–C=O) bond appears between 1700 cm^−1^ and 1750 cm^−1^. The associated peak appears at 1748 cm^−1^ for PLA and its composites, whereas the –C=O peaks of PBS (1713 cm^−1^) and PBAT (1711 cm^−1^) are shifted to lower wavenumbers, indicating a more flexible and polarizable chemical environment [92].

An increase in the shoulder (~1700–1711 cm^−1^) and in the intensity of the carbonyl (–C=O) stretching band (~1711 cm^−1^) was observed in the PBS/OP, PBS/BSG, and PBAT/BSG biocomposites compared to the corresponding neat polyesters. However, no significant shift (5–30 cm^−1^) of this band toward lower wavenumbers was detected in these materials. Therefore, no conclusive evidence of hydrogen bond formation between the hydroxyl groups (–OH) of the lignocellulosic fillers and the ester carbonyl groups (–C=O) of the polyester matrices can be drawn based on the ATR-FTIR spectral interpretation of these biocomposites [93].

The observed spectral changes are more likely attributable to the following: (i) the overlapping of carbonyl-related vibrational bands from the filler components (e.g. cellulose, lignin, proteins), and/or (ii) modifications in local polarity or the microstructural environment of the matrix.

In PBS-based composites, the increase in both the shoulder and band intensity is more pronounced for BSG than for OP, possibly due to the higher content of hydroxyl and carbonyl groups in BSG constituents (cellulose, lignin, proteins), enhancing the spectral band superposition effects. In contrast, a decrease in both shoulder and intensity is observed in PBAT/OP compared to neat PBAT, which may indicate a disruption of existing intermolecular interactions within the matrix.

Accordingly, the comparison of FTIR spectra between neat polymers and their lignocellulosic filled biocomposites does not reveal any significant appearance, disappearance, or shift in characteristic bands. This suggests a limited interaction between the matrix and the filler, with no detectable formation of specific bonds such as hydrogen bonding. The filler appears to be physically dispersed within the matrix, and the processing conditions likely resulted in an interface governed mainly by physical adhesion [94].

### 3.6. Thermogravimetric Analysis (TGA)

**Figure 8:**
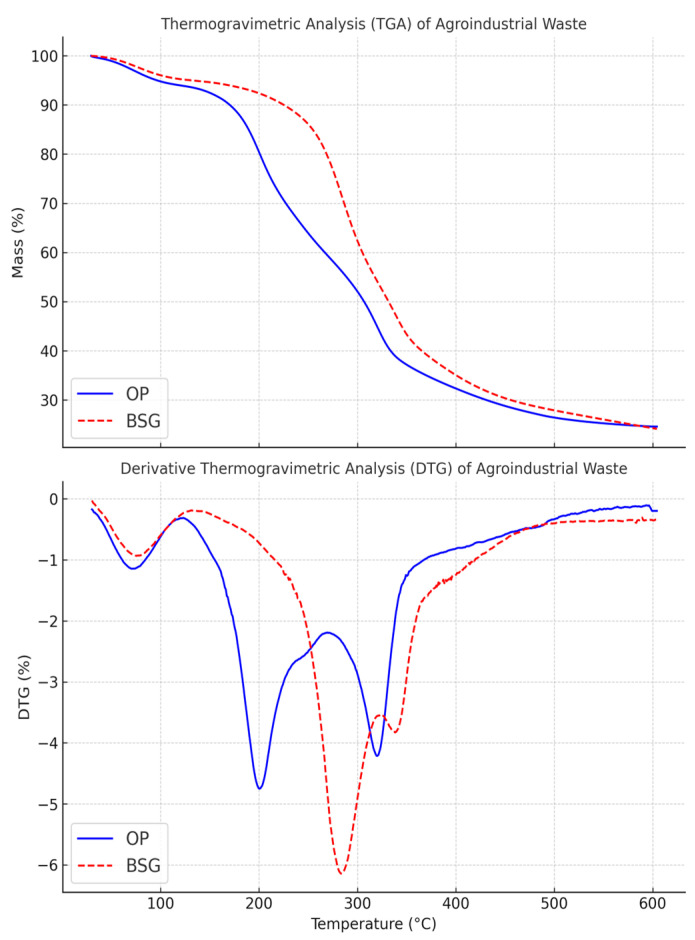
Thermograms (TG) and first derivatives of the thermograms (DTG) of the two industrial co-products (i.e., OP and BSG) obtained by thermogravimetric analysis under a non-oxidizing gas (i.e., argon).

**Figure 9:**
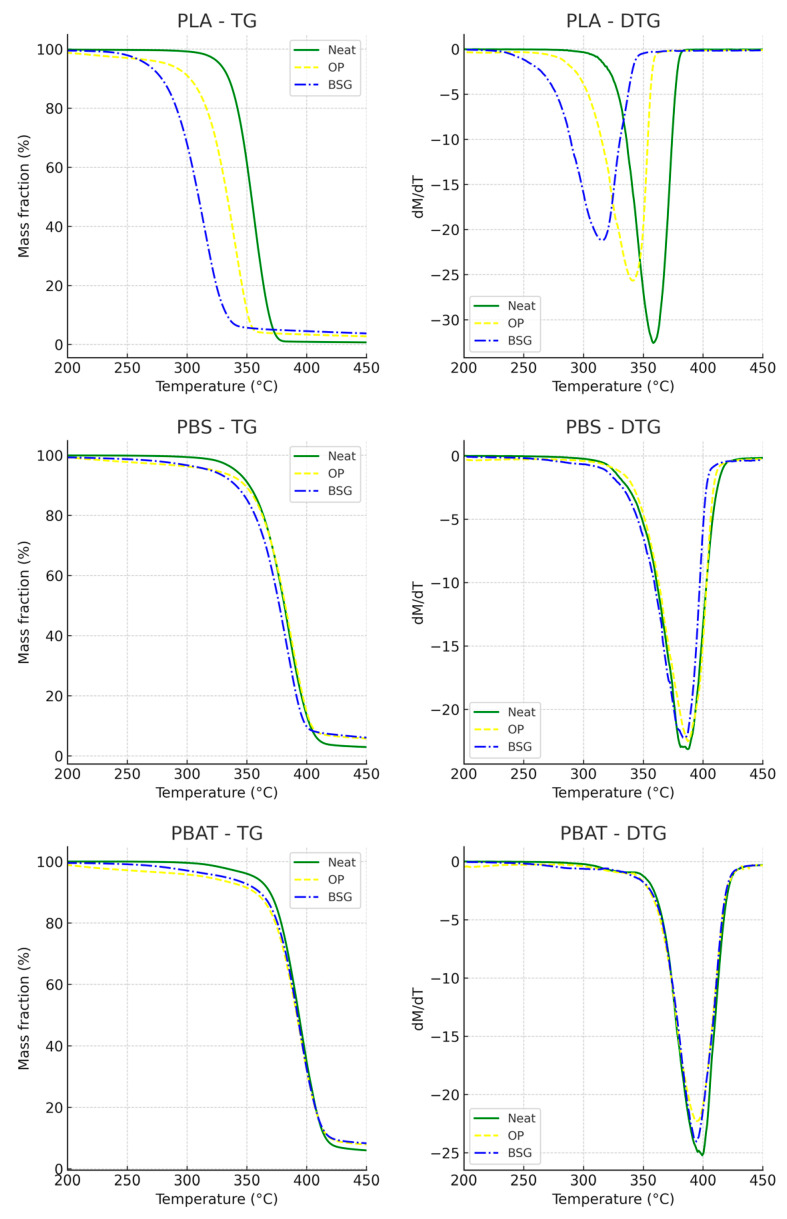
Thermograms (TG) and first derivatives of the thermograms (DTG) of shaped materials (neat polymers and biocomposites) obtained by thermogravimetric analysis under a non-oxidizing gas (i.e., argon).

The thermograms (TG) of agro-industrial by-products and biocomposites, along with their first derivative functions (DTG), are shown in Figure 8 and Figure 9, and their main parameters are summarized in Table 4. The analyses were carried out on pre-dried samples (20 ± 2 mg, Al_2_O_3_ crucible) under an argon flow (10 mL min^−1^), from 20 °C to 600 °C, at a heating rate of 10 K min^−1^.

For the two agro-industrial by-products, the curves can be divided into three distinct phases. The first phase of mass loss primarily occurs below 150 °C and corresponds to the evaporation of residual water and volatile fractions from the samples. Orange peel (OP) exhibits a higher DTG peak than brewer’s spent grain, occurring at 73 °C, which suggests that OP has a higher residual moisture content and is therefore more hygroscopic. Subsequently, a second shoulder appears, corresponding to the decomposition of light volatile fractions.

Two additional thermal decomposition peaks appear at 202.1 °C and 320.7 °C for OP, and 287.3 °C and 341.6 °C for BSG. These peaks can be attributed to the degradation of hemicellulose and cellulose, respectively, cellulose being more thermally stable than hemicellulose. The thermal decomposition of hemicellulose and cellulose occurs over different temperature ranges due to the variability in their chemical structures. For cellulose, a higher degree of polymerization, crystallinity, and larger crystallite size are associated with greater thermal stability [95,96].

A third thermal degradation is observed between 370 and 400 °C, corresponding to the non-oxidative degradation of lignin, which is thermally more stable [97]. The more pronounced shoulder for BSG in this temperature range aligns with the biochemical fractionation results, confirming that BSG contains a higher lignin content than OP.

The thermograms of the polymeric materials display a characteristic sigmoidal shape, suggesting that thermal degradation proceeds in a single step without the formation of stable intermediate compounds. The incorporation of organic fillers results in a noticeable reduction in the thermal stability of all biocomposites. This decrease is particularly significant for PLA-based biocomposites compared to those based on PBS and PBAT.

This effect is particularly evident in PLA/BSG, where the T_d5%_ parameter decreases by 18.6% (T_d5%_ for PLA/BSG: 267.2 ± 7.4 °C) compared to pure PLA (T_d5%_ for PLA: 327.3 ± 1.2 °C). Similarly, T_d5%_ for PLA/OP decreases by 13.7% (PLA/OP: 283.3 ± 4.9 °C). The same trend is observed for the temperature corresponding to the maximum thermal decomposition rate (DTG_max_) and its intensity. PLA/BSG and PLA/OP exhibit maximum mass losses of 2.12%w/°C at 316.5 ± 8.4 °C and 2.57%w/°C at 341.5 ± 1.5 °C, respectively, whereas pure PLA loses 3.26%w/°C at 358.6 ± 1.1 °C.

These results indicate that the extent and intensity of the reduction in thermal stability and DTGmax depend on the nature of the matrix–filler combination. This reflects both the impact of matrix–filler interactions and the intrinsic degradation behavior of each neat matrix, with comparisons made at equivalent filler contents (10 wt.%).

The residue at 600 °C mainly derives from the inorganic fraction of the fillers and the polyester matrix, as well as from carbonaceous chars formed under an inert atmosphere.

Carrasco et al. [90] investigated the influence of processing conditions on the thermal and mechanical properties of PLA. Although they demonstrated a positive linear relationship between molecular weight (M_w_) and the initial thermal decomposition temperature (T_d5%_) of the polymer, the slope of this relationship was relatively low, indicating a weak sensitivity of T_d5%_ to M_w_ within the studied range (162.5–212.3 kDa). A 24.5% reduction in molecular weight resulted in only a 1.8% decrease in T_d5%_. Regarding the temperature at 50% mass loss (T_d50%_), which approximates the maximum thermal decomposition temperature of PLA, no difference was observed between the 162.5 kDa and 212.3 kDa samples. This suggests that the lower molecular weights observed in our PLA-based biocomposites (PLA/BSG: M_w_ = 70.1 kDa; PLA/OP: M_w_ = 76.4 kDa; PLA: M_w_ = 89.9 kDa), resulting from an additional thermo-extrusion step, are unlikely to be the primary cause of the marked decrease in thermal stability observed in these biocomposites compared to the neat polymer.

Similar results to ours were reported by Mittal et al. [98], who observed a more pronounced decline in the thermal stability of PLA-based biocomposites containing date seed powder compared to their PBAT-based counterparts. The authors also noted greater immiscibility between the filler and the PLA matrix at high loading levels (>20%) due to poor interfacial compatibility.

Indeed, weak interfacial adhesion between organic filler particles and the polymer matrix can further exacerbate the decline in the thermal stability of biocomposites. This phenomenon is attributed to chemical, mechanical, and thermal interactions at the interface occurring during the decomposition process.

Additional evidence from the literature supports this interpretation. For instance, Ju et al. [44] developed PLA/soda lignin biocomposites (10–30 wt.%), with or without polyethylene glycol (PEG) modification. Despite prior drying and processing via twin-screw extrusion, biocomposites containing unmodified lignin exhibited a marked reduction in thermal stability under nitrogen compared to both neat PLA and the PEG-modified systems. Similarly, Manshor et al. [99] reported a significant decrease in the thermal stability of PLA/durian seed shell fiber composites (70/30 wt.%), which was mitigated by a 4 wt.% NaOH pretreatment of the fiber. This treatment reduced the filler’s hydrophilicity and improved its compatibility with the PLA matrix, highlighting the crucial role of interfacial adhesion in enhancing the thermal stability of PLA-based biocomposites. Kim et al. [44] also observed a sharp decline in thermal stability for PLA composites containing cassava or pineapple flour (70/30 wt.%), despite adequate drying procedures. The use of maleic anhydride to enhance interfacial bonding limited, but did not fully prevent, thermal degradation. Kamaludin et al. [100] similarly attributed the substantial thermal instability of their PLA/chitosan composites to poor interfacial adhesion, which promoted autocatalytic carbon–carbon bond scission and consequently lowered the decomposition temperature.

Arrigo et al. [101], for their part, attributed the accelerated thermal decomposition of PLA/biochar composites to the catalytic effect of potassium (21.6 wt.% in the biochar, corresponding to approximately 0.54 wt.% in the biocomposite containing 2.5 wt.% biochar) present in the filler. Based on potassium content data from the literature—34 mg/g in brewer’s spent grain [102] and 8.12 mg/g in orange peel [103]—our BSG and OP biocomposites may contain approximately 0.27% and 0.065% potassium by weight, respectively, potentially contributing to catalytic degradation phenomena.

For instance, weak adhesion prevents the polymer matrix from acting as a protective barrier for the filler particles, exposing them more directly to high temperatures. As a result, these filler particles decompose earlier during heating, potentially generating volatile degradation products (e.g., free radicals) that can further degrade the polymer matrix, thereby accelerating the overall thermal degradation of the biocomposite. Moreover, inhomogeneous heat distribution within the biocomposite, caused by the presence of fillers, can lead to the formation of cracks and cavities, intensifying the thermal degradation process [104,105,106].

The particularly pronounced decrease in thermal stability observed in PLA-based biocomposites compared to PBS- and PBAT-based ones highlights the stark contrast in the effect of filler incorporation depending on the nature of the polymer matrix. This difference can be attributed not only to the poor interfacial compatibility between PLA and lignocellulosic fillers such as orange peel (OP) and brewer’s spent grain (BSG), but also to the intrinsic thermal instability of PLA [90].

Furthermore, despite the higher intrinsic thermal stability of BSG compared to OP, the PLA/BSG composite exhibited lower thermal stability than PLA/OP. This discrepancy highlights that the thermal behavior of PLA-based biocomposites cannot be reliably predicted using a linear additive model. Instead, it reflects complex matrix–filler interactions that can significantly influence thermal decomposition kinetics. Poor interfacial adhesion likely contributed to this result, as suggested by the lower tensile strength of PLA/BSG compared to PLA/OP (Table 6).

Overall, these findings highlight the importance of optimizing the matrix–filler interface in the design of PLA-based biocomposites to improve their thermal stability. In contrast, PBS- and PBAT-based biocomposites appear to be less sensitive to reductions in thermal stability when incorporating untreated fillers, even without the use of compatibilization strategies.

Despite the marked reduction in thermal stability observed for certain biocomposites (i.e., PLA/OP and PLA/BSG), all formulations exhibit a maximum decomposition temperature (*T*_dpeak_) exceeding 300 °C. This threshold ensures their suitability for conventional thermoplastic processing methods (e.g., extrusion, thermoforming) as well as for end-use applications involving moderate thermal exposure, such as packaging. They can therefore be considered appropriate for the intended applications.

### 3.7. Scanning Electron Microscopy (SEM)

**Figure 10:**
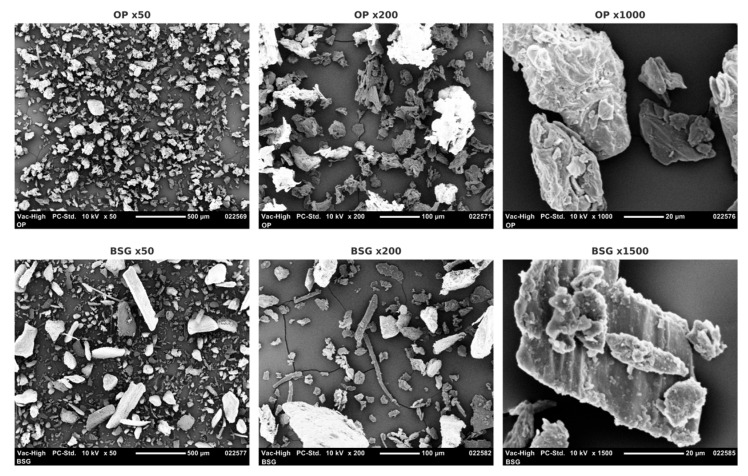
SEM images of agro-industrial by-products at magnifications of ×50, ×200, and ×1000 or ×1500.

**Figure 11:**
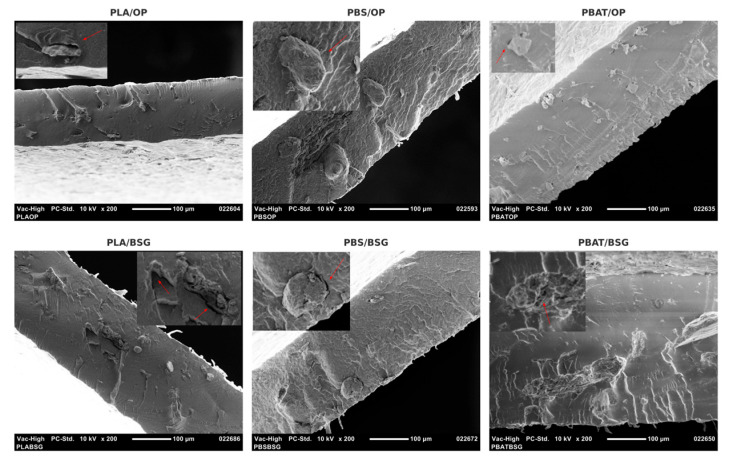
SEM images of cryo-fractured cross-sections of the biocomposites (fractured in liquid nitrogen), at ×200 magnification, including close-ups of the particle–matrix interface. Red arrows indicate selected regions of the matrix–filler interface.

**Figure 12:**
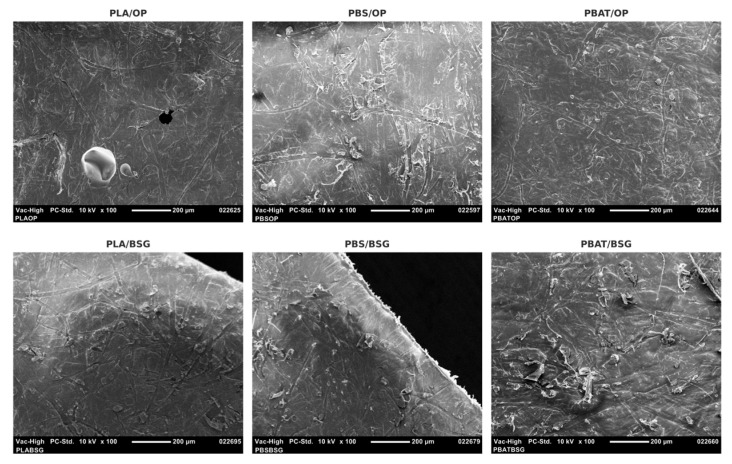
SEM images of the surface of the biocomposites at ×100 magnification.

Figure 10, Figure 11 and Figure 12 present SEM images of the agro-industrial by-products, cryo-fractured cross-sections in liquid nitrogen of the biocomposite materials, and surfaces of the resulting biocomposites, respectively. The multiscale morphological analysis reveals clear differences between orange peel (OP) and brewer’s spent grain (BSG), which are expected to influence their behaviors when incorporated into polymer matrices.

High-magnification SEM images show that orange peel (OP) particles exhibit more pronounced surface roughness and a higher density of surface asperities, along with a more regular and homogeneous macroscopic morphology compared to brewers’ spent grain (BSG) particles. These features are consistent with nitrogen physisorption results (Table 3), which indicate a higher specific surface area for OP. Increased surface roughness is generally considered an indirect indicator of greater external porosity [107,108], which may promote improved wettability within the polymer matrix and enhance interfacial adhesion. It is also commonly associated with a greater water absorption capacity of the filler [109].

In contrast, BSG particles exhibit a much smoother surface, a more heterogeneous macroscopic shape, and a coarser granulometry. This morphology suggests lower external porosity and a reduced interfacial contact area, which may limit interactions with the polymer matrix. These structural differences—also evident in the particle size distribution curves (Figure 4)—could contribute to the weaker interfacial adhesion and the reduced mechanical performance observed in BSG-based biocomposites.

Cryo-fractured cross-sections of the biocomposites reveal a slightly more pronounced interfacial void volume around filler particles in PLA-based biocomposites compared to those based on PBS and PBAT (Figure 11). This observation suggests weaker interfacial adhesion between the matrix and the filler in PLA systems, implying that the cellulosic fillers are less compatible with PLA. In contrast, filler particles in PBS- and PBAT-based biocomposites appear to be more uniformly embedded within the matrix, indicating better wetting behavior [110].

Furthermore, PLA/BSG biocomposites appear to exhibit greater filler–matrix debonding and larger interfacial void volumes compared to PLA/OP biocomposites. Such disparities may stem from the greater heterogeneity in shape and size of BSG particles, their higher average volume, lower specific surface area, and smoother surface morphology compared to OP particles—all factors that contribute to reduced wettability within the polymer matrix [111,112].

Moreover, except for the PLA/OP biocomposite, the surfaces of all biocomposites appear smooth and homogeneous, reflecting the effect of thermocompression (Figure 12). In contrast, the PLA/OP surface displays distinct dark, circular areas, which likely correspond to cavities left by missing filler particles, some reaching several hundred micrometers in diameter. In general, the thermal degradation of lignocellulosic fillers can lead to a reduction in surface polarity, thereby enhancing their compatibility with hydrophobic polymer matrices such as PLA, as previously reported [113]. However, our observations suggest that, in the case of PLA/OP, the thermopressing conditions (165 °C for 3 min, including 1.5 min at 200 bars) may have caused excessive degradation of the orange peel (OP) particles, potentially leading to fragmentation and loss of filler structural integrity.

Interestingly, such surface cavities were not observed in the PLA/BSG biocomposite, likely due to the higher thermal stability of brewer’s spent grain (BSG) (Figure 8), nor were they detected in PBS- or PBAT-based biocomposites, which were processed at a lower temperature (135 °C), potentially limiting the thermal degradation of the OP fillers during processing.

### 3.8. Differential Scanning Calorimetry (DSC)

The calorimetric parameters associated with the heat capacity of the samples are gathered in Table 5.

The glass transition temperatures (T_g_) measured during the first heating cycle show that PLA/OP (63.8 °C) and PLA/BSG (63.2 °C) are slightly lower than that of pure PLA (65.9 °C). In the second heating cycle, the T_g_ of all PLA-based materials decreases as follows: PLA (60.4 °C), PLA/OP (54.4 °C), and PLA/BSG (58 °C).

From DSC analysis, we must keep in mind that if the second heating cycle allows us to erase the thermal history of the material, the first heating cycle reveals the structural state of the material as it is used and in relation with its mechanical properties and water behavior.

**Figure 13:**
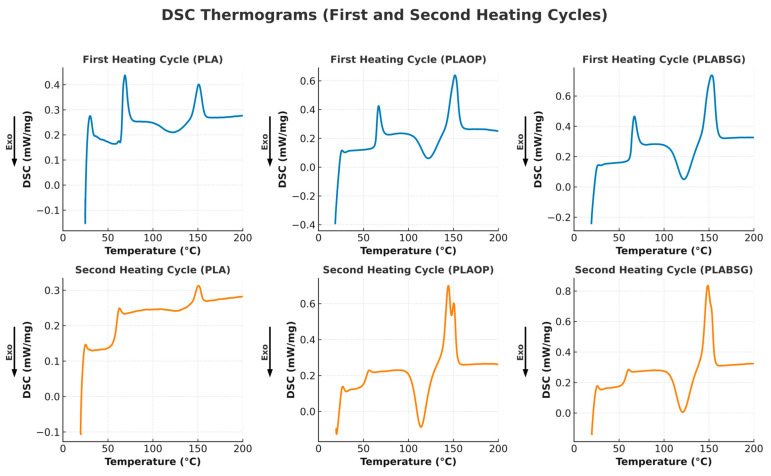
DSC thermograms of PLA-based materials for the two heating cycles.

The T_g_ peak observed during the first heating cycle resembles a melting peak (Figure 13), suggesting the occurrence of endothermic molecular relaxation due to the release of stress accumulated during the thermoforming and conditioning stages. As a result of molecular relaxation during the first heating cycle, the second heating cycle exhibits a reduction in the glass transition temperature (T_g_) of pure PLA. This decrease can be attributed to increased chain mobility following the release of internal stresses induced during processing [113], while the lower T_g_ values observed in biocomposites during the first heating cycle imply a plasticizing effect of the organic fillers on the polymer matrix. The efficiency of filler plasticization appears to be quite similar between the two types of fillers in this case, as the T_g_ of PLA/OP and PLA/BSG are close together.

During the second heating cycle, PLA/OP exhibits a significantly lower glass transition temperature (T_g_) (54.4 °C ± 1.8) than PLA/BSG (58 °C ± 1.0), indicating a stronger plasticizing effect of the OP filler. Given its lower thermal stability (Figure 8), OP likely undergoes partial degradation during the first heating, releasing volatile and/or semi-volatile compounds into the matrix. Orange peel essential oil is particularly rich in limonene (>90%), a monoterpene with a well-documented plasticizing effect on polyester matrices [114,115,116,117]. Given its relatively high boiling point (~176 °C) and thermal stability [37], a fraction may remain trapped within the filler or polymer matrix after processing at 165 °C. This residual limonene likely migrates into the PLA phase, increasing chain mobility and contributing to the observed T_g_ reduction in PLA/OP.

Thermogravimetric analysis (TGA) supports this interpretation: OP exhibited a mass loss of 13.67% between 120 and 200 °C, compared to only 2.88% for BSG. In the corresponding biocomposites, PLA/OP lost 0.73% versus 0.18% for PLA/BSG in the same temperature range. This approximately fourfold difference suggests that a substantial amount of semi-volatile compounds is retained in PLA/OP and may diffuse into the matrix, thereby reducing T_g_. A similar hypothesis was proposed by Plagiarini et al. [37], who observed a significant increase in elongation at break in PBSA/OP biocomposites (85/15, *w*/*w*) relative to neat PBSA, which they attributed to the migration of orange peel oil from the filler to the polymer matrix. Similarly, Sambudi et al. [19] also reported a plasticizing effect of orange peel in PLA-based biocomposites.

This reversible plasticizing effect is unlikely to result from residual moisture, as both PLA/OP and PLA/BSG showed nearly identical content after 24 h at 105 °C (0.89% and 0.87%, respectively). Moreover, DVS measurements showed comparable water vapor sorption for both biocomposites under storage conditions (≤50% RH). At a water activity of 0.5, the equilibrium moisture content was 7.0 × 10^−3^ g/g for PLA/BSG and 6.7 × 10^−3^ g/g for PLA/OP.

However, in the absence of direct measurements—such as the GC-MS analysis of the polyester matrix volatile profile before and after the secondary plasticization, the specific contribution of limonene or other volatile compounds cannot be conclusively established and therefore remains a working hypothesis.

In addition to this reversible effect, both composites exhibit an irreversible reduction in T_g_ after the first heating cycle. This likely results from physical plasticization due to the presence of dispersed filler particles, which increase free volume and reduce intermolecular interactions (e.g., van der Waals forces) between polymer chains [19]. The resulting increase in chain mobility contributes to the overall thermal softening of the material.

Altogether, these findings emphasize the significant role of volatile compound retention in agro-industrial fillers and its impact on the thermal properties of PLA-based biocomposites. The marked T_g_ depression observed in PLA/OP compared to PLA/BSG highlights the dual plasticizing behavior of orange peel: as a physical plasticizer and as a source of entrapped semi-volatiles that act as reversible chemical plasticizers under thermal conditions.

Concerning the degree of crystallinity, as expected, PLA and its biocomposites remain in an amorphous state (*χ* = ~1%); the presence of organic fillers has no nucleating effect on the PLA matrix. This can be readily explained by the relatively high D-lactide content (4%), which disrupts the stereochemical regularity required for the formation of well-ordered crystalline structures [118]. This aligns with well-established findings in the literature, as lignocellulosic fillers are generally known to exhibit low to moderate nucleating ability compared to inorganic nucleating agents (e.g., talc). Such nucleating potential is typically insufficient to significantly enhance crystallinity when the D-lactide content is high.

**Figure 14:**
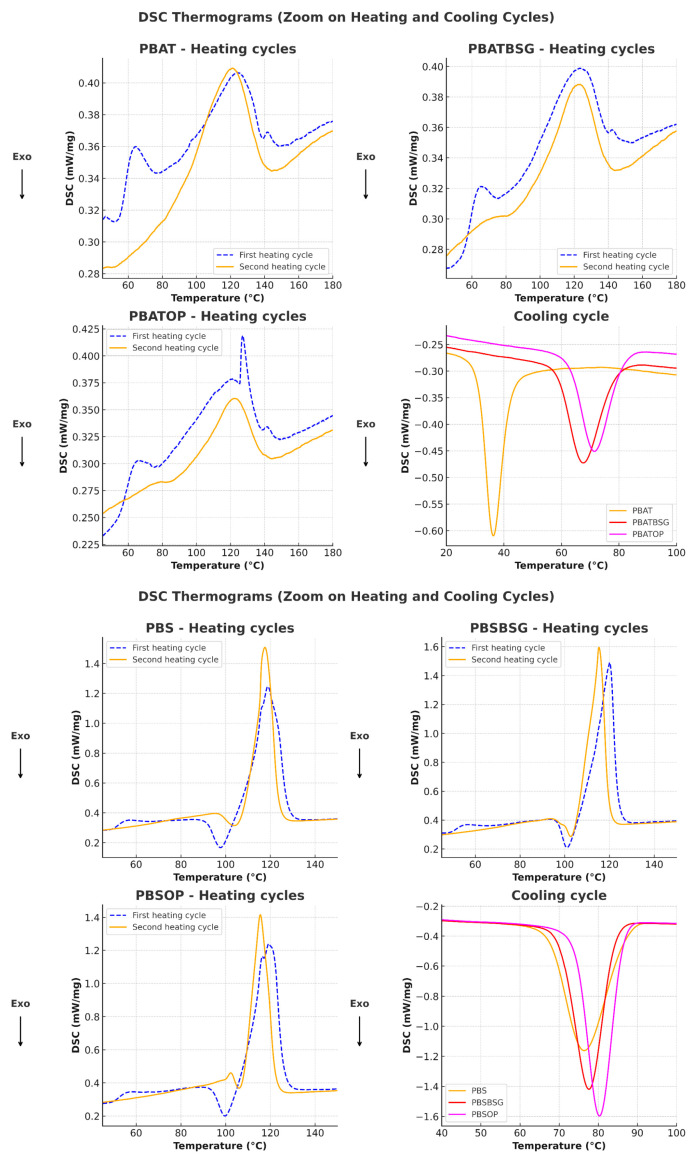
DSC thermograms of PBS- and PBAT-based materials, highlighting the first and second heating cycles as well as the cooling cycle.

In the case of PBS-based materials, the thermograms obtained during the first heating cycles exhibit two distinct peaks: a small peak around 55 °C and a main peak within the 95–130 °C range (Figure 14). In contrast, the thermograms of PBAT-based materials reveal the presence of multiple endothermic peaks in the temperature range of 45–145 °C. However, the thermograms associated with the second heating cycles of both polyester-based materials show only a single main peak.

In general, the crystallization of polymers is a multi-step process that progresses through crystal nucleation, followed by primary and secondary crystallization [119].

The intermediate endothermic peaks observed during the first heating cycle can plausibly be attributed to secondary crystallization within certain amorphous regions of the polymers, leading to the formation of smaller, imperfect crystals with lower thermal stability [120,121]. Androsch et al. [122] have already observed this phenomenon on PBS fragments subjected to different crystallization temperatures.

Secondary crystallization is favored by rapid cooling [123], as applied during our material fabrication. The plastic sheets were swiftly removed from the thermo-press and compressed between metal plates to accelerate cooling. This process may have led to the formation of metastable crystallites with varying thermal stability. These smaller crystallites, with a reduced specific surface area and higher free energy, require less thermal energy to melt, resulting in lower melting temperatures [123].

Regarding the crystallinity levels of PBS-based biocomposites, no significant changes were observed compared to pure PBS during the first heating cycle, indicating, as with PLA-based materials, that the filler does not act as a nucleating agent. In the second heating cycle, the disappearance of the intermediate endothermic peak observed in PBS-based materials suggests the elimination of the metastable fraction. The crystallinity levels measured are consistently lower than those of the first heating cycle, implying that molecular reorganization has increased the amorphous fraction in all PBS-based materials.

For PBAT-based materials, metastable fractions represent ~7.2 to 10.9% of the material mass, compared to ~1.3 to 2% for PBS-based materials, indicating that secondary crystallization is more pronounced in PBAT.

In contrast to PLA and PBS-based biocomposites, for PBAT-based biocomposites incorporating organic fillers (OP and BSG), the crystallinity levels calculated during the first heating cycle are consistently higher than those of pure PBAT, both for the main peak and for all peaks combined. This would suggest that the organic fillers act as heterogeneous nucleating agents, facilitating the formation of crystallites during the initial cooling [98,124]. This result is consistent with the higher crystallization temperatures T_c_ of the biocomposites compared to pure PBAT indicating that organic fillers promote the early onset of crystallite formation. During processing, interactions between the filler and the matrix likely reduced the activation energy required to initiate crystallization [125].

Xu et al. [126] similarly reported a reduction in the supercooling point of the polymer for thermopressed PBAT/corn stover biocomposites, attributing this effect to the aromatic structure of lignin present in the by-product, which promotes the mixed crystallization of the butylene terephthalate (BT) units. In contrast, in our case, orange peel (OP) contains only a small amount of lignin (0.7% ± 0.1) compared to corn stover (~15%), suggesting that different mechanisms may be at play.

In the second heating cycle, the crystallinity of the biocomposites is reduced by about half compared to the first cycle, while pure PBAT maintains nearly constant crystallinity, as expected. This reduction can be attributed to the elimination of metastable crystalline fractions, as indicated by the presence of a single main melting peak, without their reorganization into more stable crystallites, as occurs with the pure polymer. This suggests that, during the second heating cycle, the fillers inhibited the molecular reorganization process, preventing these metastable fractions from transforming into more stable forms.

### 3.9. Tensile Strength Tests

The main parameters of the tensile strength tests [ISO 527-3] are presented in Table 6. All biocomposites exhibit lower tensile strength values compared to their corresponding neat polymers. This reduction is statistically significant at the 5% threshold (Tukey-HSD test) for all biocomposite materials. Among them, PBS/OP displays the smallest decrease, with a reduction of 25.0%.

The incorporation of brewers’ spent grain (BSG) leads to a greater reduction in the tensile strength of biocomposite films. Specifically, PLA/BSG decreases by 53.8%, PBS/BSG by 28.9%, and PBAT/BSG by 42.3%, whereas the reductions observed with orange peel (OP) are comparatively lower, reaching 42.8% for PLA/OP, 25.0% for PBS/OP, and 26.3% for PBAT/OP. This more pronounced decrease in the case of BSG-based composites can be attributed to the poorer matrix-filler compatibility. The larger particle size, heterogeneous distribution, smoother surface, and lower specific surface area of BSG may promote particle decohesion and aggregation, ultimately leading to the formation of interfacial voids and weak zones within the material.

The mechanical properties of biocomposite materials are strongly influenced by the morphology, size, and distribution of the filler particles, as well as the strength of interfacial adhesion between the filler and the polymer matrix. The weak interfacial adhesion of the filler creates stress concentration zones, thereby reducing the tensile strength. This could explain the more significant loss of tensile strength observed in PLA/BSG (−53.8%) and PBAT/BSG (−42.3%).

Among PLA-based biocomposites, the decrease in tensile strength is particularly pronounced, reaching 42.8% with OP and 53.8% with BSG. This phenomenon could be explained by the intrinsic rigidity of PLA, which limits its ability to absorb and distribute mechanical stress in areas of poor adhesion [127]. Additionally, the very low compatibility of PLA with hydrophilic fillers results in a degraded interface, promoting the formation of microcracks under mechanical stress [128].

For PBS and PBAT-based composites, although reductions in tensile strength are also observed, they remain comparatively moderate, suggesting a better matrix-filler compatibility. In this regard, PBS/OP exhibits a decrease in only 25.0%, whereas PBAT/OP shows a slightly greater reduction of 26.3%.

The incorporation of fillers also leads to significant changes in the Young modulus. PBS-based biocomposites exhibit an increase in stiffness, 11% for PBS/OP and 23.6% for PBS/BSG. A similar trend is observed for PBAT-based biocomposites, where PBAT/OP increases by 21.1% and PBAT/BSG by 39%. These results confirm that the inclusion of rigid fibrous fillers increases the stiffness of ductile polymer matrices such as PBS and PBAT. This effect is primarily due to the physical presence of dispersed stiff particles, which limit the mobility of polymer chains, and—particularly in the case of PBAT—may also be associated with an increase in matrix crystallinity [129,130].

In contrast, the Young’s modulus of PLA-based biocomposites decreases, with PLA/OP showing a reduction of 5.9% and PLA/BSG a decrease of 16.5%. This reduction is likely due to the disruption of the rigid structure of the PLA matrix, caused by the incorporation of organic fillers that are relatively more compliant than the polymer itself.

The more fibrous structure of BSG accounts for the greater increase in Young’s modulus observed for PBS/BSG (+23.6%) and PBAT/BSG (+39%) compared to their respective PBS/OP and PBAT/OP biocomposites. Indeed, the high cellulose content of these fillers likely contributes to the overall stiffening of the ductile and viscoelastic PBS and PBAT matrices, owing to the inherently high rigidity of cellulose. The simultaneous increase in stiffness and decrease in tensile strength is a well-documented phenomenon in biocomposites containing hydrophilic cellulosic fillers and a hydrophobic polymer matrix, in the absence of any compatibilization strategy [131].

Elongation at break values also decrease significantly in the biocomposites. The reductions observed for PLA-based composites reach 40.0% for PLA/OP and 46.7% for PLA/BSG, while for PBS-based biocomposites, PBS/OP decreases by 43.7% and PBS/BSG by 41.4%. However, for PBAT-based biocomposites, the incorporation of BSG results in a more pronounced decrease in elongation at break, reaching 31.9%, compared to a reduction of 17.5% for PBAT/OP. This difference could be attributed to the more fibrous structure of BSG, which increases stiffness and reduces the extent of plastic deformation prior to fracture.

Regarding the impact of using a partially moist lignocellulosic filler during processing, Berthet et al. (2015) [67] demonstrated that wheat straw fibers with up to 14.2% moisture (wet basis) promoted the hydrolysis of the PHBV matrix during extrusion, resulting in a significant reduction in molecular weight. This structural alteration can negatively affect mechanical properties. In addition, the presence of a water layer at the fiber surface may hinder interfacial bonding between functional groups of the filler and the matrix. However, Berthet et al. [67] observed no significant difference in the mechanical performance of composites made with dry or moist fibers. This was attributed to a plasticizing effect of PHBV oligomers formed during matrix hydrolysis, which may have helped preserve the mechanical properties. Overall, moisture sensitivity depends strongly on the matrix type, filler content, and actual moisture level at processing, and should therefore be evaluated on a case-by-case basis.

Optimizing filler dispersion and improving interfacial adhesion could help mitigate mechanical losses while leveraging the increased stiffness for targeted applications.

### 3.10. Surface Energy

The Owens–Wendt model was applied to calculate the surface free energy (SFE) of our samples, including polyester fragments and biocomposites, as well as their dispersive and polar components (Table 7). These calculations were based on static contact angle measurements performed with three different liquids: water, diiodomethane, and ethylene glycol. The primary reason for considering the SFE of thermoplastic materials lies in its role in determining friction and wear properties [132]. Surface interactions govern critical phenomena such as adhesion, wettability, and material compatibility in various industrial applications [133].

Adhesion refers to the bonding of one material to another, a phenomenon driven by a variety of molecular interactions that are generally weak, such as hydrogen bonds and van der Waals forces. However, their intensity can vary depending on the chemical nature and topography of the contacting surfaces. In the food packaging sector, SFE plays a crucial role as it affects the interaction between food and packaging, thereby influencing product quality and preservation [134]. A low SFE reduces food adhesion to the surface, which is often desirable to facilitate cleaning and prevent contamination. Conversely, strong adhesion is essential in sealing areas to prevent leaks and external contamination.

The obtained results indicate that PLA fragments exhibit a relatively low SFE of 39.8 mJ·m^−2^, which is lower than that of PBS (45.5 mJ·m^−2^) and PBAT (46.5 mJ·m^−2^). The difference stems from the higher dispersive components of PBS and PBAT compared to PLA, which increases their total surface free energy while maintaining a similar capacity for dipolar interactions with polar liquids such as water.

Regarding biocomposites, the incorporation of cellulosic organic fillers alters the SFE differently depending on the polymer matrix. In general, the polar component increases significantly due to the presence of hydroxyl (–OH) groups in the mono-, di-, or polysaccharide of the fillers. In the packaging sector, this increase in surface polarity may enhance adhesion with other materials or functional layers, such as water-based inks [135].

Meanwhile, the dispersive component slightly decreases due to the reduction in available surface areas for van der Waals interactions, a consequence of surface occupation by hydrophilic functional groups [136].

The results show that the polar component of surface energy is significantly higher for PLA/BSG (20.1 mJ·m^−2^) than for PLA/OP (5.2 mJ·m^−2^), and similarly for PBAT/BSG (14.4 mJ·m^−2^) compared to PBAT/OP (6.7 mJ·m^−2^). In contrast, the values for PBS/OP (6.0 mJ·m^−2^) and PBS/BSG (2.1 mJ·m^−2^) remain relatively close and are inverted. This suggests that a greater amount of polar hydroxyl groups is exposed to the surface of PLA/BSG and PBAT/BSG biocomposites, which may reflect a higher degree of filler particle exposure and hence a less homogeneous dispersion of BSG within these two matrices. This phenomenon can be attributed to the lower interfacial compatibility between the polymer and the lignocellulosic filler, resulting in the greater surface agglomeration of filler particles [137].

This interpretation is supported by mechanical data, which reveal a more pronounced decrease in tensile strength for PLA/BSG (16.3 MPa) and PBAT/BSG (12.3 MPa) compared to PLA/OP (20.2 MPa) and PBAT/OP (15.7 MPa). Conversely, PBS/OP (22.8 MPa) and PBS/BSG (21.6 MPa) exhibit comparable values, indicating better interfacial adhesion in the PBS/BSG system.

Surface topography modifications, such as roughness, spatial organization, and structural arrangement, could also contribute to the observed variations in contact angles, although no direct measurements of these parameters were available in this study. It is well established that the incorporation of cellulosic fillers into polymer matrices can induce the micro structuring of the surface, thereby influencing liquid interactions. However, the extent of this effect strongly depends on the dispersion and distribution of the fillers within the matrix, parameters that would require specific measurements for further analysis [138].

In summary, the results indicate that the incorporation of cellulosic fillers profoundly alters the balance between the polar and dispersive components of the SFE in biocomposites. While this modification can be advantageous for applications requiring good wettability and adhesion, it could also increase susceptibility to microbial biofilm formation due to enhanced polarity and water retention capacity [139]. A deeper understanding of surface interactions could be leveraged to develop biocomposites with tailored properties for specific applications, such as active packaging or biodegradable films with controlled wettability.

### 3.11. Water Vapor Sorption (DVS)

The investigation of water vapor sorption capacity and the associated diffusion mechanisms of polymers and their biocomposites is of significant interest in the biomedical and food packaging sectors [140,141]. These materials must withstand varying hygroscopic conditions, as excessive water absorption can alter their mechanical properties, increase oxygen (O_2_) permeability, and reduce their functional lifespan [142]. For instance, in the food industry, water transfer from the external environment into the packaging can lead to premature food spoilage, raising concerns about food safety [143]. However, in the context of mesophilic biodegradation, higher sorption in this range can be advantageous, as it promotes moisture penetration, polymer chain scission, and microbial colonization.

**Figure 15:**
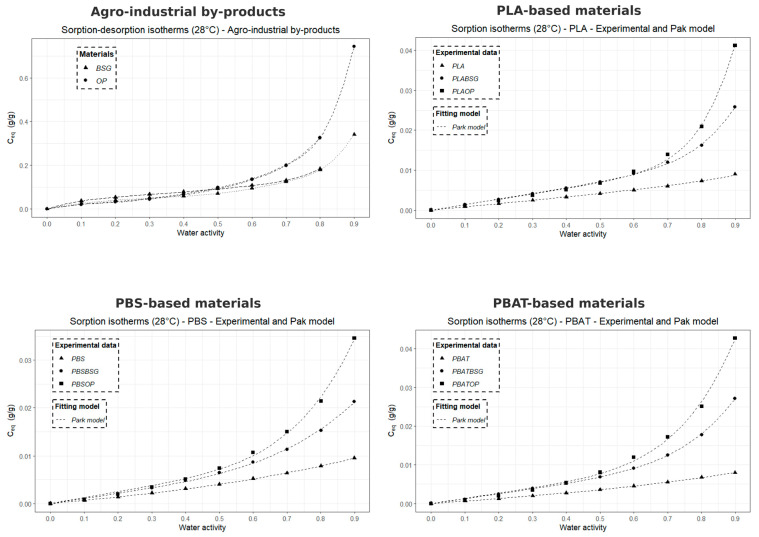
Water sorption–desorption isotherms at 28 °C for agro-industrial by-products (OP, BSG), and water sorption isotherms at 28 °C for PLA-, PBS-, and PBAT-based materials and their biocomposites. Experimental data and non-linear mechanistic Park model fitting are shown for polymer-based materials only. C_eq_: equilibrium concentration of permeant molecules (g/g) at a given water activity (*a_w_*).

The water vapor sorption isotherms of agro-industrial byproducts and materials are presented in Figure 15. Both cellulosic fillers exhibit moderate sorption at low water activity levels (*a_w_* ≤ 0.4), followed by a characteristic inflection point typical of type II isotherms when water activity exceeds 0.5 (*a_w_* ≥ 0.5). This transition is commonly observed in porous materials rich in hydrophilic functional groups.

At a high-water activity level of 0.9, the sorption capacity of orange peel (C_eq_ = 0.7429 g/g) is significantly higher than that of brewers’ spent grain (C_eq_ = 0.3409 g/g). The high-water vapor sorption capacity of organic fillers is primarily attributed to the strong polarity of their chemical groups, leading to hydrogen bonding with water molecules [144]. Additionally, the high internal porosity of these byproducts plays a key role in enhancing their water retention capacity.

Since FTIR analyses revealed a higher intensity in the 3200–3600 cm^−1^ region for brewers’ spent grain (BSG), corresponding to O–H stretching vibrations, this indicates a greater abundance of chemically hydrophilic functional groups compared to orange peel (OP). Therefore, the higher water sorption capacity observed for OP particles is likely not of chemical origin, but rather attributable to their physical structure—such as increased internal and external porosity and greater surface roughness—relative to BSG particles.

As previously discussed, SEM observations showed that orange peel (OP) particles have significantly higher surface roughness than brewers’ spent grain (BSG) particles (Figure 10), which appear much smoother. This is consistent with our BET surface area results, showing that orange peel particles have a higher specific surface area (0.4207 m^2^/g) than brewers’ spent grain (0.3838 m^2^/g). In lignocellulosic materials, such a roughness is often considered an indirect indicator of greater external porosity [107,108], which facilitates early-stage water uptake by enhancing access to surface-active sites.

However, to rigorously confirm the hypothesis that the porosity (both internal and external) of the fillers accounts for the 2.17-fold higher sorption capacity of OP compared to BSG at *a_w_* = 0.9, internal porosity measurements should be conducted. These should include total pore volume and pore size distribution, as these properties can significantly influence water vapor sorption behavior [109].

The sorption isotherms of neat polymers (PLA, PBS and PBAT) exhibit a linear profile, indicating a typical sorption behavior of low-hydrophilicity materials. At a water activity level of 0.9, the water vapor sorption capacity varies little among the three polymers (C_eq_ (g/g): PLA = 0.089; PBS = 0.095; PBAT = 0.081) and remains low (<1%). The sorption isotherms of biocomposites; however, display a convex shape at high water activities (*a_w_* ≥ 0.5). The incorporation of organic fillers leads to a significant increase in water vapor sorption capacity, facilitating water penetration into the material. A portion of this water accumulates locally in the cavities of the filler particles as well as at the particle/matrix interface.

Biocomposites containing orange peel exhibit higher water vapor sorption capacity than those containing brewers’ spent grain, which is consistent with the inherent sorption properties of the fillers themselves.

### 3.12. Mixing Law

**Figure 16:**
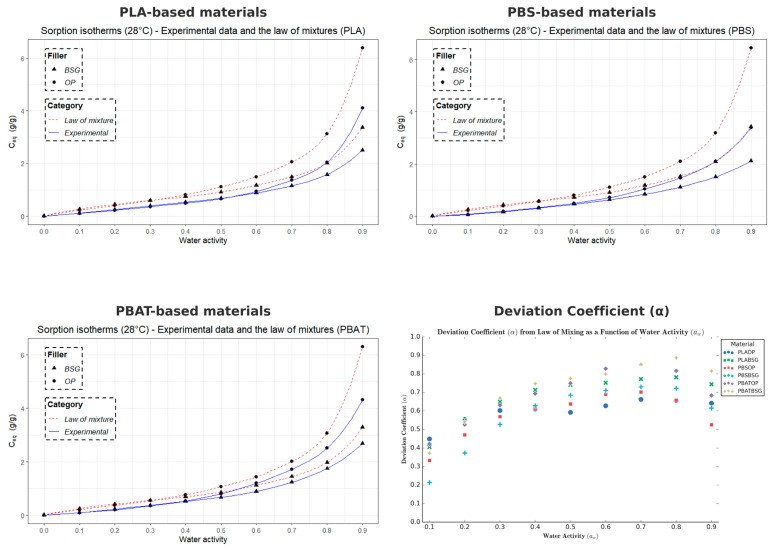
Water sorption isotherms at 28 °C for PLA-, PBS-, and PBAT-based biocomposites (experimental data vs. theoretical values predicted by the linear additive mixing law), and corresponding deviation coefficients (α). Ceq: equilibrium concentration of permeant molecules (g/g) at a given water activity (aw). The deviation coefficient (α) quantifies the ratio between experimental and predicted sorption ($\alpha =\frac{C_{Experimental}}{C_{Mixing\;law}}$), as a function of water activity.

To better understand the contribution of each component to the water vapor sorption capacity of biocomposites, a mixing law (Equation (15)) was applied to theoretically estimate the water vapor sorption of the biocomposites, based on the mass fractions of both constituents present.

(15) $$C_{composite}=W_{filler}C_{filler}+W_{matrix}C_{matrix}$$

where $C_{composite}$ is the water concentration (g/g) in the biocomposite material, $W_{filler}\;and$ $C_{filler}$ are the mass fraction and water concentration of the biocomposite filler, respectively, and the same applies to $W_{matrix}C_{matrix}$ for the polymer matrix. These water molecule concentrations were calculated for each water activity level (0.1–0.9) and are presented in Figure 16.

The results show that the mixing law systematically overestimates the permeant molecule concentration within the biocomposite over the entire range of water activities.

Accordingly, a deviation factor (α) from the mixing law was calculated for each water activity level and each material, using the following equation:

(16) $$\alpha =\frac{C_{Experimental}}{C_{Mixing\;law}}$$

where $C_{Experimental}$ and $C_{Mixing\;law}$ law refer to the water molecule concentrations at a given water activity, determined experimentally and predicted theoretically by the linear additive mixing law model, respectively.

The deviation coefficients for all biocomposite materials are plotted as a function of water activity in Figure 16. All values are below 1 (α < 1), indicating that the actual water sorption in the biocomposites is lower than that predicted by a linear additive combination of the individual constituents. The deviation from the mixing law predictions is more pronounced at low water activities and tends to decrease as the water activity increases.

Thus, the water vapor sorption behavior of our biocomposites cannot be explained by a simple additive contribution of their individual components. Within the biocomposite structure, interactions between the filler and the polymer matrix negatively affect the sorption behavior by reducing the amount of water present in the material at each level of relative humidity tested.

To gain deeper insight into the water vapor sorption behavior of the biocomposites, a non-linear mechanistic modeling approach was applied using the Park model. This model was employed to identify the underlying sorption mechanisms and to quantitatively assess the contributions of distinct sorption modes, namely Langmuir-type adsorption, Henry’s law dissolution, and aggregation-related uptake.

### 3.13. Modeling of Sorption Isotherms (Park’s Model)

The five parameters of the mechanistic Park model, fitted to our experimental water vapor sorption isotherms data at 28 °C for plastic materials (Figure 15), are presented in Table 8. Our sorption isotherms were successfully modeled using the five-parameter Park model, as reflected by the deviation modulus values of our samples (*E*’ < 10%) [61], except for PBATOP which shows a slight deviation (+3.43%).

The five parameters calculated from the model provide insight into the underlying mechanisms of water vapor sorption within the samples [145,146,147,148]. At low water activity levels (*a_w_* ≤ 0.1), the Langmuir sorption mode, represented by its two terms ($A_{l}\;and\;b_{l})$, is predominant. For moderate water activity (0.1 < *a_w_* < 0.5), Henry-type sorption becomes dominant, characterized by the slope coefficient ($k_{h})$ of the corresponding isotherm. At high water activity levels (*a_w_* > 0.5), water molecules tend to aggregate due to increasing water-water interactions [149]. The parameters $K_{a}$ and n respectively reflect this phenomenon through the rate constant of aggregate formation and the average number of permeant molecules per formed cluster.

The values of *A_l_* for the three neat polymers are very similar. The biocomposites generally show a decrease in this parameter, except for PLA/OP which has a value comparable to PLA. This suggests a depletion in the concentration of accessible polar sorption sites on the surface of biocomposite materials. In other words, the incorporation of a filler disrupts water molecule sorption on the polymer matrix surface. This phenomenon has previously been observed in PLA-based nanocomposite films containing modified nano clays [145]. One possible explanation is that the high polarity of the filler particles at the material surface induces strong cohesion between water molecules via hydrogen bonding between water and the filler [145]. This would be consistent with the increase in the b_L_ constant affinity when fillers are incorporated in the polymer matrix.

In the case of PLA/OP, the thermomechanical degradation of the filler during the thermopressing step—evidenced by the presence of cavities observed in SEM surface images—may explain why this biocomposite is the only one that does not exhibit any change in the Langmuir component parameters compared to the neat PLA matrix.

Although cavities are observed on the surface of PLA/OP, their limited number suggests that most orange peel (OP) particles remain embedded in the matrix after thermopressing. Therefore, the unchanged value of the *A_l_* parameter is unlikely to result from the absence of filler, but rather from a loss of polarity at the particle surface.

At the pressing temperature of 165 °C, OP likely undergoes partial thermochemical degradation, leading to the breakdown of hydrophilic functional groups (e.g., hydroxyl and carboxyl). As a result, the surface of the filler becomes less polar and less interactive with water vapor. Although still present, the degraded particles no longer significantly interfere with the polar sorption sites of the PLA matrix.

This contrasts with other biocomposites, especially those containing BSG, where the thermally stable fillers retain their polarity and compete with the matrix for water sorption, resulting in a decrease in the *A*_l_ parameter.

Regarding Henry’s law, which mainly governs sorption behavior at intermediate water activity levels, the $k_{h}$ constant is found higher for biocomposites than for neat polymers, indicating greater solvent solubility within these samples. The presence of the filler enhances the random sorption of water molecules throughout the amorphous regions and within the filler particles themselves. As expected, biocomposites containing orange peel exhibit a higher value for this constant compared to those containing brewers’ spent grain. In the case of PLA/OP, it is noteworthy that the $k_{h}$ value is equivalent to that of PLA/BSG (1.35 × 10^−2^), and noticeably higher than that of neat PLA (8.16 × 10^−3^). This suggests that the partial thermomechanical degradation of the filler at the biocomposite surface, resulting from the pressing temperature of 165 °C, did not impair the random sorption of water within the material’s structure.

At high water activity levels (*a_w_* > 0.5), the presence of a filler amplifies water aggregation within the matrix for all three polyesters studied. The values of the $K_{a}$ constant and the n parameter are both higher than those of neat polymers, indicating a greater affinity of the solvent within the biocomposite and a larger number of molecules forming aggregates. The $K_{a}$ parameter is higher for OP biocomposites compared to BSG biocomposites, suggesting greater ease in aggregate formation. This trend may be related to the higher porosity of OP particles.

Finally, based on the analysis of deviation coefficients (α) from the linear mixing law presented in the previous section, the strongest deviations from ideal additivity between the matrix and the filler were observed at low to intermediate water activity levels (0.1–0.5), and became less pronounced at higher water activities (*a_w_* > 0.5). These results suggest that matrix–filler interactions primarily interfere with Langmuir-type and Henry-type sorption mechanisms, which are predominant in this lower *a_w_* range.

In particular, the intrinsic capillarity of the two lignocellulosic fillers (BSG and OP) may promote the early accumulation of water molecules within their internal porosity, thereby diverting water away from the amorphous phase of the polymer. This would result in localized heterogeneous sorption occurring at relatively low water activities, contributing to an earlier onset of aggregation-type sorption within the biocomposites. Consequently, the relative contribution of Henry-type dissolution would be reduced under these conditions.

### 3.14. Diffusion Coefficients

**Figure 17:**
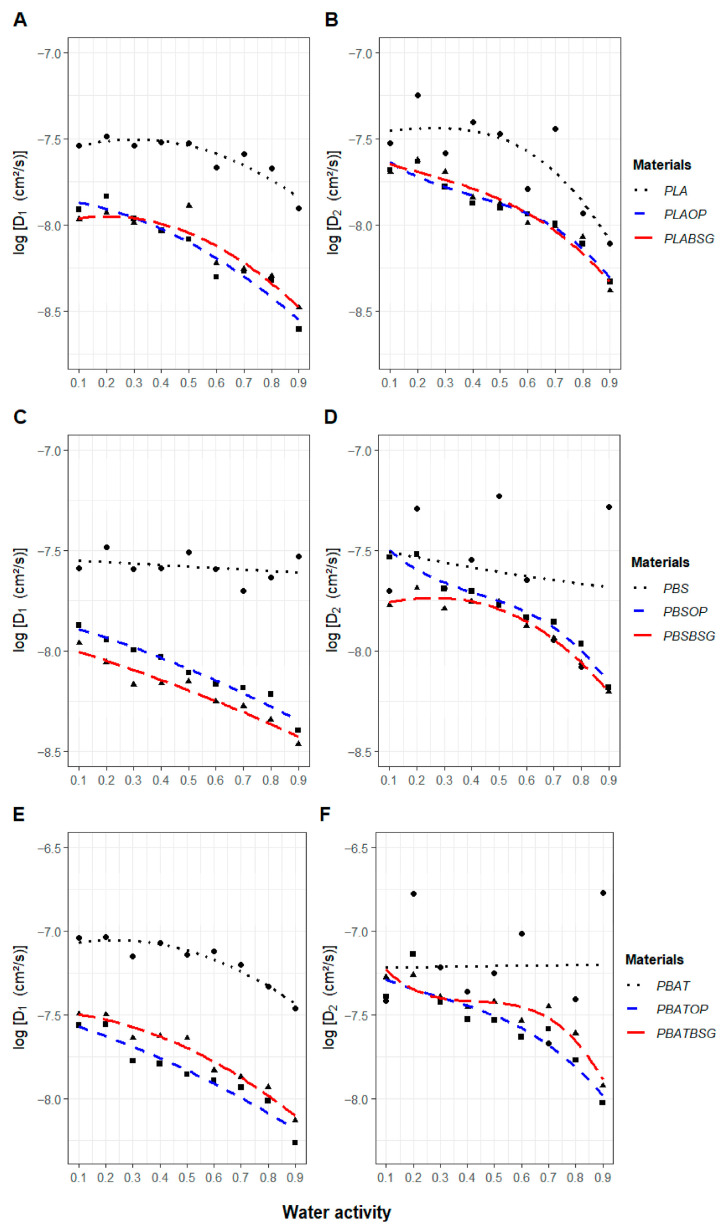
Diffusion coefficients (D_1_ and D_2_) of the materials determined from water vapor sorption kinetics, represented on a semi-logarithmic scale. (**A**) D_1_ for PLA-based materials; (**B**) D_2_ for PLA-based materials; (**C**) D_1_ for PBS-based materials; (**D**) D_2_ for PBS-based materials; (**E**) D_1_ for PBAT-based materials; (**F**) D_2_ for PBAT-based materials. Experimental data are represented by squares, circles, and triangles, while regression curves are represented by lines.

The diffusion coefficients (*D*_1_ and *D*_2_) of the samples, calculated from the first and second half-sorption kinetics, are plotted on a semi-logarithmic scale as a function of water activity (0.1–0.9) in Figure 17. These two parameters indicate the rate of moisture penetration at different humidity levels, a key factor for packaging and other moisture-sensitive applications.

Here, *D*_1_ represents the initial rate of water uptake during the first half of the sorption process (*M*(t)/*M*_eq_ < 0.5). In contrast, *D*_2_ corresponds to the diffusion rate in the latter stages (*M*(t)/*M*_eq_ > 0.5), when moisture penetrates deeper into the material and approaches steady-state conditions.

The presence of an organic filler decreases the water diffusivity (*D*_1_) within the biocomposites during the first half-sorption phase. This reduction reflects the classic tortuosity effect associated with the dispersion of the filler within the polymer matrix, which increases the path length for diffusing molecules, thereby reducing the permeability of the materials [146]. The magnitude of this reduction appears to be largely independent of the type of filler used, suggesting a comparable dispersion/exfoliation level between the different biocomposites.

At high water activity, all materials except PBS exhibit a significant decrease in *D*_1_ values, which corresponds to the aggregation of water molecules. This effect is attributed to the increasing size of diffusing species, which lose mobility as they form clusters [149,150]. However, the PBS-based sample does not show such a decrease in water diffusivity during the first half-sorption phase (*D*_1_), suggesting a low degree of water molecule aggregation and, consequently, little or no reduction in diffusivity. This hypothesis is supported by the lower values of the $K_{a}$ and n parameters in Park’s model for PBS, compared to the materials (PLA and PBAT) that exhibit a diffusion slowdown (*D*_1_) at high water activity.

For neat polymers, the diffusion coefficients *D*_1_ and *D*_2_ fall within the same range of values over the water activity range. As previously suggested [145], two opposing phenomena likely contribute to this behavior over longer timescales (∆*mt/*∆*meq* ≥ 0.5). The first is the plasticizing effect of water molecules, which act as a plasticizer and can increase water diffusion rates. The second is the aggregation of water molecules within the matrix, which can slow down diffusion.

However, during the second half-sorption phase, the diffusion coefficient (*D*_2_) increases for the biocomposite materials. The observation is that *D*_2_ > *D*_1_ can be attributed to the swelling of the matrix due to the substantial water uptake by the fillers. This swelling leads to two main effects: (i) the plasticization of the matrix, which enhances the mobility of polymer chains by breaking secondary bonds, thereby contributing to a higher diffusion rate, and (ii) a reduction in the tortuosity effect observed for *D*_1_, as the increased distance between filler particles diminishes the physical barrier effect. As a result, the mobility of the chains increases, accelerating the diffusion within the matrix, while the tortuosity effect is reduced, allowing water to follow a more direct and less convoluted path through the structure.

### 3.15. Mean Cluster Size (MCS)

**Figure 18:**
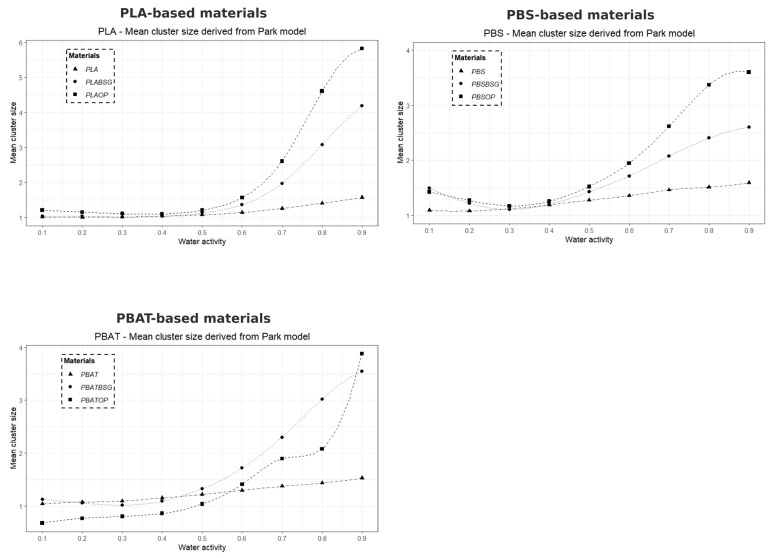
Evolution of the mean cluster size (MCS) as a function of water activity for PLA-, PBS-, and PBAT-based materials and their biocomposites, as estimated from the Park model fitting.

The mean cluster sizes determined by using Park’s model (${MCS}_{Park}$) for our samples, calculated from experimental water vapor sorption isotherm data, are shown as a function of water activity in Figure 18. From a physical standpoint, the MCS parameter provides an estimate of the average number of water molecules aggregated into clusters within the material at a given water activity. Higher values indicate stronger water–water interactions and more extensive clustering, whereas lower values suggest a predominance of water–material interactions.

The hypothesis of cluster formation is commonly proposed when an increase in water solubility (as a function of water activity) is accompanied by a decrease in water diffusivity, which implies an increase in the diameter of permeant molecules [151]. At this stage of discussion, it should be remembered that the *n* parameter represents the water cluster size deduced from the Park model and highly depends on the fitting at highest water activities while the MCS reveals the water cluster size but depending on the water concentration in the material (linked to the water activity). From that, *n* is supposed to be close to the MCS at the highest water concentrations. Therefore, the values of *n* and the MCS are in the same order of magnitude, and the same trends are observed as PLA-based composites are characterized by higher water cluster size than PBS and PBAT-based ones (Table 8).

The estimation results of ${MCS}_{Park}$ indicate that cluster formation within neat polymers is significantly limited compared to their biocomposites. Clustering primarily occurs at the filler particles and at the interface between the filler particles and the polymer chains, as evidenced by the sorption isotherms of the agro-industrial by-products. Clustering begins at a water activity of 0.4 for PBS and PBAT and at 0.5 for PLA and their corresponding biocomposites. The increase in water cluster size with increasing water concentration (increase in water activity) aligns with the observed reduction in diffusion.

On average, clusters in PLA-based biocomposites are larger than those in PBS and PBAT biocomposites. While the cluster sizes within the neat polymers do not differ significantly (~1.5 for *a_w_* = 0.9), PLA-based biocomposites likely have a greater porosity, facilitating more extensive cluster expansion within the structure. This porosity is likely in the form of microvoids at the polymer-filler interface due to the very weak compatibility between the two components, as previously highlighted in the thermogravimetric analysis (TGA), and mechanical tensile results.

At this temperature (28 °C), PLA remains in a glassy state (*T_g_* ~ 60 °C), unlike PBS and PBAT. In this state, polymer chain segments no longer have sufficient energy to undergo rapid segmental movements and are thus “frozen” on experimentally accessible timescales [152]. Consequently, the low mobility of these chains prevents them from reaching their theoretical equilibrium volume. The difference between the volume of a glassy polymer and its theoretical equilibrium volume is known as excess volume, which manifests as micro voids with volumes on the order of a few hundred cubic angstroms [153].

### 3.16. Hysteresis

**Figure 19:**
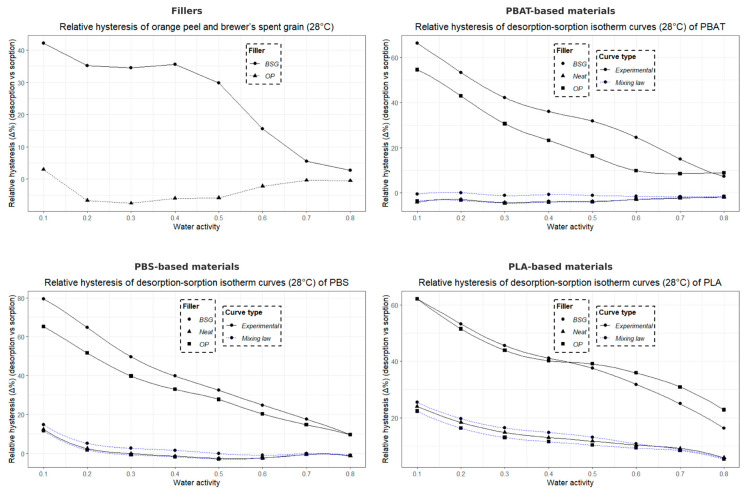
Evolution of the relative hysteresis (Δ%) of water sorption–desorption isotherms as a function of water activity for agro-industrial by-products (BSG, OP), and for biocomposites based on PLA, PBS, and PBAT.

The relative desorption–sorption hysteresis in water uptake for all materials (fillers, neat polymers, and biocomposites) was calculated at each water activity level (0.1–0.8) using the following equation:

(17) $$Relative\;Hysteresis\left(\%\right)=\frac{C_{desorption}-C_{sorption}}{C_{sorption}}\times 100$$

where *C_desorption_* and *C_sorption_* represent the equilibrium moisture contents (g/g) during desorption and sorption, respectively. These results are plotted as a function of water activity in Figure 19.

Sorption-desorption hysteresis occurs when the kinetic evolution of a material’s water content during the sorption phase diverges from that observed during the subsequent desorption step, resulting in a non-superimposition of the two curves [154]. In packaging applications, moisture cycling can induce repeated sorption–desorption events. A pronounced hysteresis may lead to gradual dimensional changes due to matrix swelling and relaxation during sorption, followed by incomplete recovery during desorption. This can compromise dimensional stability over time.

For a given water activity, if *C_desorption_* > *C_sorption_*, the relative hysteresis is positive, indicating that more water is retained during the desorption phase than was initially adsorbed during sorption. Conversely, if *C_desorption_* < *C_sorption_* the hysteresis is negative, suggesting that water is more readily released during desorption.

Overall, orange peel exhibited slightly negative relative hysteresis across the water activity range, while brewer’s spent grain (BSG) showed a markedly positive hysteresis that progressively decreased at higher water activities (*a_w_* > 0.5). This indicates that during desorption, orange peel retains slightly less water than it initially adsorbed during sorption, whereas BSG retains more, reflecting fundamental differences in water–material interactions.

In fiber-rich, cellulosic materials like BSG, the coupling between sorption and swelling is known to induce positive hysteresis. Indeed, sorption–desorption hysteresis is a well-documented phenomenon in hydrophilic polymers that swell upon water uptake [154]. During desorption, the contractive forces of the polymer network are often insufficient to fully expel adsorbed water molecules [155]. As reported by Chen et al. [155], in soft nanoporous polymers such as cellulose, hysteresis does not stem solely from hydrogen bonding, but rather from a coupling between sorption and the structural reorganization of the porous matrix. Swelling alters both the size and connectivity of the pores, resulting in distinct energy landscapes between the adsorption and desorption phases. Specifically, in lignocellulosic matrices, water molecules form hydrogen bonds with cellulose chains, disrupting inter-chain hydrogen bonding and inducing swelling, which subsequently exposes additional hydrophilic sites. Upon desorption, following the structural rearrangement of the composite at high humidity, the newly formed water–cellulose interactions—stronger than water–water interactions—tend to trap water molecules more effectively, making their release more difficult. This results in partial water retention and a pronounced positive hysteresis in cellulosic materials such as BSG.

In contrast, orange peel shows slightly negative or near-zero hysteresis across the tested water activity range (Figure 19). This can be attributed to its chemical composition, dominated by water-soluble compounds (e.g., mono- and disaccharides, organic acids; WSC: 80.2 ± 0.4%) and low structural polysaccharide content.

Neat polymers, on their part, exhibit contrasting behaviors depending on their physical state. PLA, which remains in a glassy state (*T* < *T_g_*) shows moderate relative hysteresis (~20% at low water activity), which gradually decreases with increasing relative humidity but remains positive (*C_desorption_* > *C_sorption_*). This hysteresis likely results from the rigidity of the polymer chains, which limits segmental mobility and hinders efficient water desorption. In contrast, PBS and PBAT polyesters, which are in a rubbery state (*T* > *T_g_*), exhibit weak or even slightly negative hysteresis (*C_desorption_* < *C_sorption_*), suggesting a more balanced absorption/desorption dynamic. This result is consistent with the findings of Pantani et al. [156], who reported no significant hysteresis for PLA fragments subjected to sorption-desorption isotherms near their glass transition temperature (58 °C). When PLA transitions from a glassy to a rubbery state, the increased flexibility of its polymer chains enables the faster relaxation of internal stresses induced by water adsorption. Consequently, the polymer chains can reorganize and release adsorbed water molecules more easily, reducing the gap between the adsorption and desorption cycles [157].

In contrast, biocomposites exhibit consistently higher hysteresis than the neat polymer, irrespective of the water activity level or the nature of the incorporated filler. To elucidate the contribution of the filler to this enhanced hysteresis behavior, we modeled the relative hysteresis of our materials using a linear additive mixing law (Figure 19). In all cases, the experimental relative hysteresis of the biocomposites exceeded the value predicted by the model, which remained very close to that of the neat polyester. This indicates that the interaction between the filler and the polymer matrix significantly alters the desorption behavior of the biocomposites compared to the individual components, leading to a substantial increase in water retention during desorption relative to sorption. Although this hysteresis decreases with increasing relative humidity, it consistently remains higher than that of both the neat polymer and the isolated filler.

We propose that water uptake by the hygroscopic filler induces volumetric expansion, thereby exerting mechanical stress on the surrounding polymer matrix. This stress may disrupt secondary interactions such as hydrogen bonding, relax the polymer network, and consequently expose additional sorption sites. As a result, more water can be retained during desorption than was initially adsorbed, contributing to the pronounced hysteresis observed in the biocomposites. Indeed, when swelling is induced by water uptake, non-Fickian relaxations associated with plasticization disrupt secondary bonds between macromolecular chains, leading to morphological alterations in the polymer structure [157]. These phenomena are particularly pronounced in amorphous or semi-crystalline polymers, where swelling results in the localized plasticization of amorphous domains [158]. As a result, more polar sites become accessible for water interaction during desorption than during sorption, owing to a temporal lag between chain relaxation and the structural recovery of the polymer matrix.

Since PLA remains below its glass transition temperature (*T_g_*), the matrix stays in a rigid, glassy state with limited chain mobility. As a result, it is less susceptible to plasticization, and structural swelling primarily leads to the accumulation of internal stresses, promoting interfacial debonding and microcrack formation rather than the flexible accommodation of water [159]. In contrast, PBS and PBAT, which are in a more flexible rubbery state at 28 °C (T > T_g_), undergo both swelling and plasticization. Although the underlying mechanisms differ across polymer matrices, they result in similarly positive and comparable relative hysteresis values.

The swelling–plasticization phenomenon is widely recognized for its detrimental impact on the mechanical performance of biocomposites. Differential swelling between the polymer matrix and the fibers generates internal stresses that may lead to the formation of voids or microcracks at the filler–matrix interface. This compromises interfacial adhesion and initiates failure mechanisms such as debonding, delamination, and microcracking [160], which can be irreversible even after drying. These structural damages are typically associated with reduced tensile strength and increased brittleness [159].

## 4. Conclusions

This study highlights the distinct impact of incorporating organic fillers derived from agro-industrial waste (orange peel and brewers’ spent grain) into PLA-, PBS-, and PBAT-based biocomposite materials, in the absence of any compatibilization strategy between the matrix and the filler. The macroscopic behavior of the resulting biocomposites is strongly influenced by the physicochemical and morphological properties of the fillers—such as particle size distribution, surface roughness, and biochemical composition—as well as by the physical state of the polymer matrix. Whether the matrix is rigid and glassy (i.e., PLA) or rubbery at ambient temperature (i.e., PBS and PBAT) directly affects chain mobility and governs matrix–filler interactions.

OP exhibited a smaller and more homogeneous particle size with a higher surface area and roughness, while BSG showed greater heterogeneity in shape and size and higher lignin/cellulose content, contributing to distinct filler–matrix interactions, both mechanically and thermally. These differences notably influenced water sorption behavior. Significant deviations from the linear additive mixing rule were observed at low to intermediate water activities (0.1 ≤ aw ≤ 0.5), indicating non-ideal sorption driven by filler-induced effects. The Park model was subsequently applied to describe the underlying mechanisms—particularly water clustering. All biocomposites exhibited marked positive hysteresis, contrasting with the opposite trends observed for the fillers alone (OP: slightly negative; BSG: positive). This was attributed to filler-induced matrix swelling, which exposes new polar sorption sites and alters the sorption–desorption energy landscape.

Water diffusivity decreased in the presence of fillers, with a more pronounced reduction observed during the initial half-sorption stage. This suggests an increased resistance to water transport, likely due to a tortuosity effect induced by physical obstruction. Additionally, filler-specific effects were observed on the extent of water aggregation and mean cluster sizes, reflecting the fillers’ role as water retention zones and their influence on the microstructure of the sorbed phase. OP also acted as a dual plasticizing agent in PLA, both physically and chemically, via the release of semi-volatiles (e.g., limonene) under thermal conditions. Conversely, BSG led to higher matrix stiffening in PBS and PBAT biocomposites.

Overall, these results provide valuable insight into the distinct effects of OP and BSG in PLA, PBS, and PBAT—particularly regarding plasticization, stiffening, water sorption, diffusion, and hysteresis—offering guidance for compatibilization strategies aimed at developing biocomposites with tailored properties for specific packaging applications.

This “*Part A*” study forms the first step of a broader research framework. *Part B* will focus on evaluating the biodegradation of these biocomposites under mesophilic composting conditions, to link their functional properties to their end-of-life behavior and better assess their environmental relevance in realistic scenarios.

Finally, the main challenge remains optimizing biocomposite formulations to align environmental performance, economic viability, and the functional property requirements of the materials. This will require quantitative economic assessments and life cycle analyses (LCA) based on realistic usage scenarios and robust methodologies.

## Figures and Tables

**Table 1:** Main properties of the polymers (PLA, PBS, and PBAT) in pellet form used in this study, including melt flow index (MFI), density, grade, and supplier information. MFI values are expressed in g/10 min, measured at 190 °C under a load of 2.16 kg. All properties are provided by the supplier.

Polymers	Grade	MFI	Density (g/cm^3^)	Supplier
PBS	PBE 003	4–6	1.26	Natureplast (France)
PLA (amorphous)	PLE 005-A	3	1.24	Natureplast (France)
PBAT	PBE 006	4–6	1.26	Natureplast (France)

**Table 2:** Mass flow rate (g/min) measured under real extrusion conditions for PLA-, PBS-, and PBAT-based biocomposites. CV: Coefficient of Variation, calculated as CV = (σ/μ) × 100.

Materials	Mass Flow Rate (g/min)	CV (%)
PLA	6.62 ± 0.14	2.1
PLA/OP	4.06 ± 0.11	2.7
PLA/BSG	5.36 ± 0.49	9.1
PBS	8.04 ± 0.12	1.5
PBS/OP	4.97 ± 0.50	10.1
PBS/BSG	5.79 ± 0.23	4.0
PBAT	6.64 ± 0.00	0.1
PBAT/OP	3.70 ± 0.39	10.5
PBAT/BSG	3.98 ± 0.36	9.0

**Table 3:** Elemental composition (C: carbon, N: nitrogen) and structural components (Cel: cellulose, Hem: hemicellulose, WSC: water-soluble compounds, Lig/Cu: lignin/cutin fraction), and BET specific surface area of dried fillers (orange peel: OP, brewer’s spent grain: BSG).

Materials	C (%)	N (%)	Cel (%)	Hem (%)	WSC (%)	Lig/Cu (%)	BET SA (m^2^/g)
OP	42.3 ± 0.2	0.9 ± 0.04	15.3 ± 0.3	3.9 ± 0.4	80.1 ± 0.2	0.7 ± 0.1	0.4207 ± 0.0079
BSG	46.7 ± 0.3	3.4 ± 0.4	49.0 ± 0.5	24.6 ± 0.4	14.4 ± 0.1	12.1 ± 0.1	0.3838 ± 0.0079

**Table 4:** Molecular Weight Distribution and Thermal Degradation Properties: Number-average (*M*_n_) and weight-average (*M_w_*) molecular weights (eq PMMA), polydispersity index (PI = *M*𝓌/*M*_n_), thermal degradation temperatures at 5% mass loss (*T_d5%_*) and degradation peak (*T*_dpeak_), and residue at 600 °C (%), obtained from SEC and TGA analyses of neat and filled PLA, PBS, and PBAT.

Materials	M_n_ (g/mol)	M_w_ (g/mol)	PI	T_d5%_ (°C)	T_dpeak_ (°C)	Residue at 600 °C (%)
PLA	43.200	88.900	2.08	327.3 ± 1.2	358.6 ± 1.1	0.53 ± 0.1
PLA/OP	37.300	76.400	2.05	283.3 ± 4.9	341.5 ± 1.5	2.09 ± 0.5
PLA/BSG	35.300	70.100	1.99	267.2 ± 7.4	316.5 ± 8.4	3.05 ± 0.8
PBS	54.000	146.700	2.72	340.7 ± 5.0	388.6 ± 6.7	2.24 ± 0.2
PBS/OP	53.300	143.000	2.68	322.5 ± 0.4	388.4 ± 0.9	4.70 ± 0.4
PBS/BSG	52.100	133.600	2.56	319.4 ± 2.2	384.0 ± 1.2	4.74 ± 0.7
PBAT	59.700	151.000	2.53	356.5 ± 0.1	399.6 ± 0.8	4.41 ± 0.1
PBAT/OP	57.300	140.700	2.46	314.9 ± 6.0	394.5 ± 0.9	5.90 ± 0.6
PBAT/BSG	50.500	120.100	2.38	331.8 ± 5.6	394.9 ± 0.6	6.24 ± 0.9

**Table 5:** Thermal Parameters from DSC: Main thermal parameters obtained by differential scanning calorimetry (DSC). T_g_: glass transition temperature (first and second scans); T_m_: melting temperature (principal peak); T_c_: crystallization temperature; ΔH_c_: enthalpy of crystallization; χ: degree of crystallinity from different calculations.

Materials	T_g_ (°C) 1st Scan	T_g_ (°C) 2nd Scan	T_m_ (°C) (Principal Peak)	T_c_ (°C)	ΔH_c_ (J/g)	χ (%) 1st Scan (Principal Peak)	χ (%) 1st Scan (Multi Peaks)	χ (%) 2nd Scan
PLA	65.9 ± 1.0	60.4 ± 1.0	151.0 ± 1.0	–	–	–	–	–
PLA/OP	63.8 ± 1.0	54.4 ± 1.8	Two peaks	–	–	–	–	–
PLA/BSG	62.3 ± 3.3	58.0 ± 1.0	151.1 ± 3.2	–	–	–	–	–
PBAT	–	−26.6 ± 1.0	121.6 ± 1.0	36.0 ± 1.0	−18.1 ± 5.0	6.0 ± 5.0	13.2 ± 5.0	14.2 ± 5.0
PBAT/OP	–	−27.1 ± 1.0	122.9 ± 1.0	71.3 ± 1.0	−13.7 ± 5.0	11.1 ± 5.0	19.4 ± 5.0	7.8 ± 5.0
PBAT/BSG	–	−27.1 ± 1.1	122.9 ± 1.0	67.7 ± 1.0	−14.5 ± 5.0	9.4 ± 5.0	22.0 ± 5.0	8.7 ± 5.0
PBS	–	–	117.4 ± 1.0	77.4 ± 1.4	−61.3 ± 5.0	54.2 ± 5.0	55.4 ± 5.0	48.1 ± 5.0
PBS/OP	–	−45.5 ± 1.0	115.6 ± 1.0	80.3 ± 1.0	−58.8 ± 5.0	53.1 ± 5.0	54.6 ± 5.0	47.2 ± 5.0
PBS/BSG	–	−41.9 ± 1.0	116.3 ± 1.1	79.8 ± 1.9	−58.4 ± 5.0	52.0 ± 5.0	54.0 ± 5.0	45.7 ± 5.0

**Table 6:** Physical and Mechanical Properties of Neat and Filled Bioplastics: Film thickness, density, tensile modulus (|E|), tensile strength, and elongation at break of neat and filled PLA, PBS, and PBAT-based films. Superscript letters (^a^, ^b^, ^c^) indicate statistically different groups (*p* < 0.05) for each property.

Materials	Thickness (µm)	Density (g/cm^3^)	|E| (MPa)	Tensile Strength (MPa)	Elongation at Break (%)
PLA	266 ± 57	1.327 ± 0.003	2546 ± 97 ^a^	35.3 ± 4.5 ^a^	1.5 ± 0.2 ^a^
PLA/OP	198 ± 37	1.335 ± 0.001	2394 ± 125 ^a^	20.2 ± 2.3 ^b^	0.9 ± 0.1 ^b^
PLA/BSG	192 ± 50	1.370 ± 0.001	2126 ± 135 ^a^	18.3 ± 1.5 ^b^	1.0 ± 0.2 ^b^
PBS	219 ± 53	1.368 ± 0.005	615 ± 16 ^c^	30.4 ± 1.5 ^a^	8.7 ± 1.2 ^a^
PBS/OP	219 ± 49	1.356 ± 0.002	683 ± 30 ^b^	22.8 ± 1.5 ^b^	4.9 ± 0.4 ^b^
PBS/BSG	205 ± 46	1.391 ± 0.004	760 ± 29 ^a^	21.6 ± 2.0 ^b^	5.1 ± 0.8 ^b^
PBAT	385 ± 77	1.298 ± 0.002	80.5 ± 4.0 ^c^	21.3 ± 3.2 ^a^	601.3 ± 17.9 ^a^
PBAT/OP	282 ± 60	1.332 ± 0.001	97.5 ± 1.6 ^b^	15.7 ± 1.0 ^b^	495.9 ± 39.1 ^b^
PBAT/BSG	316 ± 44	1.316 ± 0.001	112.0 ± 1.4 ^a^	12.3 ± 1.1 ^c^	408.8 ± 49.9 ^c^

**Table 7:** Surface Wettability and Energy Parameters of PLA, PBS, PBAT and Their Biocomposites: Values of static contact angles measured with a goniometer on water (θ_w_), diiodomethane (θ_dii_), and ethylene glycol (θ_eg_), and surface energies calculated using the Owens–Wendt–Rabel–Kaelble (OWRK) method. γ_s_: total surface energy; γ_s_^p^: polar component; γ_s_^d^: dispersive component.

Materials	θ_w_ (°)	θ_dii_ (°)	θ_eg_ (°)	γ_s_ (mJ/m^2^)	γ_s_^p^ (mJ/m^2^)	γ_s_^d^ (mJ/m^2^)
PLA	73.8 ± 4.1	38.1 ± 4.2	73.7 ± 0.3	39.8	1.6	38.2
PLA/OP	62.0 ± 17.6	39.3 ± 3.0	62.1 ± 6.9	40.6	5.2	35.4
PLA/BSG	37.1 ± 8.5	41.9 ± 1.5	0.0	56.1	20.1	36.1
PBS	69.4 ± 1.9	13.9 ± 4.6	65.3 ± 0.7	45.5	1.4	44.1
PBS/OP	55.4 ± 7.1	29.3 ± 6.2	57.3 ± 1.1	45.1	6.0	39.2
PBS/BSG	71.6 ± 3.9	28.0 ± 2.7	61.2 ± 3.6	43.3	2.1	41.1
PBAT	75.7 ± 2.0	0.0	66.8 ± 1.4	46.5	0.5	46.0
PBAT/OP	58.1 ± 3.4	50.1 ± 5.5	71.5 ± 2.6	35.5	6.7	28.8
PBAT/BSG	0.0	0.0	46.6 ± 6.0	56.9	14.4	42.5

**Table 8:** Parameters of the Park Model Applied to Water Vapor Sorption Isotherms: Parameter values of the Park model applied to water vapor sorption isotherms of polymer samples (*a_w_* = 0.1–0.9). A_l_: Langmuir capacity constant; b_l_: Langmuir affinity constant; k_H_: Henry-type solubility coefficient; Ka: aggregation equilibrium constant; n: average number of water molecules per cluster; MCS: mean cluster size (at *a_w_* = 0.9); E′: mean percentage deviation from the model.

Materials	A_l_	b_l_	k_H_	K_a_	n	MCS (a_w_ = 0.9)	E′ (%)
PLA	1.74	4.01 × 10^−5^	8.16 × 10^−3^	2.38 × 10^−3^	5.1	1.57	0.98
PLA/OP	1.75	4.04 × 10^−5^	1.35 × 10^−2^	7.33 × 10^−2^	8.77	5.82	8.57
PLA/BSG	6.25 × 10^−2^	1.30 × 10^−2^	1.32 × 10^−2^	3.01 × 10^−2^	7.82	4.19	2.18
PBS	1.74	1.87 × 10^−6^	6.73 × 10^−3^	4.62 × 10^−3^	2.74	1.59	1.42
PBS/OP	5.72 × 10^−2^	1.07 × 10^−2^	1.18 × 10^−2^	3.87 × 10^−2^	5.17	3.60	9.94
PBS/BSG	5.84 × 10^−2^	1.15 × 10^−2^	9.99 × 10^−3^	1.81 × 10^−2^	4.24	3.11	8.14
PBAT	1.74	4.23 × 10^−4^	5.53 × 10^−3^	3.23 × 10^−3^	2.85	2.15	0.89
PBAT/OP	5.92 × 10^−2^	1.53 × 10^−2^	1.25 × 10^−2^	5.56 × 10^−2^	5.7	4.72	13.43
PBAT/BSG	5.90 × 10^−2^	1.52 × 10^−2^	1.19 × 10^−2^	2.91 × 10^−2^	5.89	3.85	2.76

## Data Availability

The data presented in this study are openly available in Zenodo, at https://doi.org/10.5281/zenodo.15017889.
